# Following the adverse outcome pathway from micronucleus to cancer using H2B-eGFP transgenic healthy stem cells

**DOI:** 10.1007/s00204-020-02821-3

**Published:** 2020-07-22

**Authors:** Bastian Niklas Hölzel, Kurt Pfannkuche, Bernhard Allner, Hans Thomas Allner, Jürgen Hescheler, Daniel Derichsweiler, Henner Hollert, Andreas Schiwy, Julia Brendt, Michael Schaffeld, Alexander Froschauer, Petra Stahlschmidt-Allner

**Affiliations:** 1 GOBIO GmbH, Institute for Ecology of Waters and Applied Biology, Scheidertalstraße 69a, 65326 Aarbergen, Hesse Germany; 2 Institute for Molecular Physiology, Johannes Gutenberg-University Mainz, Johann-Joachim Becher-Weg 7, 55122 Mainz, Rhineland Palatinate Germany; 3 Medical Faculty, Center for Physiology and Pathophysiology, University of Cologne, Robert Koch Str. 39, 50923 Cologne, North Rhine-Westphalia Germany; 4 Evolutionary Ecology and Environmental Toxicology, Goethe University Frankfurt Biologicum, Max-von-Laue-Straße 13, 60323 Frankfurt am Main, Hesse Germany; 5 Institute for Environmental Research (Bio V), RWTH Aachen University, Worringerweg 1, 52062 Aachen, North Rhine-Westphalia Germany; 6 Faculty of Biology, Applied Biology, Technische Universität Dresden, Zellescher Weg 20b, 01069 Dresden, Saxony Germany; 7 EWOMIS GmbH, Schießstraße 26c, 63486 Bruchköbel, Hesse Germany

**Keywords:** Micronucleus test, Non-destructive live-cell imaging technology, AOP for malign transformation

## Abstract

In vitro assessment of genotoxicity as an early warning tool for carcinogenicity mainly relies on recording cytogenetic damages (micronuclei, nucleoplasmic bridges) in tumour-derived mammalian cell lines like V79 or CHO. The forecasting power of the corresponding standardised test is based on epidemiological evidence between micronuclei frequencies and cancer incidence. As an alternative to destructive staining of nuclear structures a fish stem cell line transgenic for a fusion protein of histone 2B (H2B) and enhanced green fluorescent protein (eGFP) was established. The cells are derived from koi carp brain (KCB) and distinguish from mammalian culturable cells by non-tumour-driven self-renewal. This technology enables the analysis of genotoxic- and malign downstream effects in situ in a combined approach. In proof-of concept-experiments, we used known carcinogens (4-Nitroquinoline 1-oxide, colchicine, diethylstilbestrol, ethyl methanesulfonate) and observed a significant increase in micronuclei (MNi) frequencies in a dose-dependent manner. The concentration ranges for MNi induction were comparable to human/mammalian cells (i.e. VH-16, CHL and HepG2). Cannabidiol caused the same specific cytogenetic damage pattern as observed in human cells, in particular nucleoplasmic bridges. Metabolic activation of aflatoxin B1 and cyclophosphamide could be demonstrated by pre-incubation of the test compounds using either conventional rat derived S9 mix as well as an in vitro generated biotechnological alternative product ewoS9R. The presented high throughput live H2B-eGFP imaging technology using non-transformed stem cells opens new perspectives in the field of in vitro toxicology. The technology offers experimental access to investigate the effects of carcinogens on cell cycle control, gene expression pattern and motility in the course of malign transformation. The new technology enables the definition of Adverse Outcome Pathways leading to malign cell transformation and contributes to the replacement of animal testing. Summary: Complementation of genotoxicity testing by addressing initiating events leading to malign transformation is suggested. A vertebrate cell model showing "healthy" stemness is recommended, in contrast to malign transformed cells used in toxicology/oncocology.

**Electronic supplementary material:**

The online version of this article (10.1007/s00204-020-02821-3) contains supplementary material, which is available to authorized users.

## Introduction

Substances impairing the integrity of the genome of somatic and generative cells and thereby causing cancer are called genotoxins. The underlying mode of action comprises chromosomal damages, mutations, gene expression alterations or aneuploidy, which are related to carcinogenic transformation in somatic cells and to heritable genetic disorders in germline cells (Bonassi et al. 2007). The potential of chemicals to induce genetic changes implicated in carcinogenesis and birth defects has triggered the development of several methods for genotoxicity assessment (Dearfield et al. 2002). It is accepted that no single test is suited to detect all varieties of genetic damages and regulatory guidelines recommend batteries of tests to assess cytogenetic damages (Ouedraogo et al. 2012). These batteries comprise bacterial and eukaryotic test systems monitoring direct interaction with the DNA molecule (AMES, UMU-chromotest and COMET assay) and test systems additionally capable of detecting effects on chromosomes, spindle apparatus and cell cycle control (sister chromatid exchange, micronucleus test and chromosome aberration test) (Dearfield et al. 2002). Recently, a further type of cytogenetic damages, nucleoplasmic bridges, came into focus in cell culture-based test systems. This specific damage has been found to be induced by cannabinoids in addition to the main endpoint micronuclei (Russo et al. 2019).

The state of the art in vitro test systems—whether performed with bacteria or eukaryotic cells—show only reduced metabolic competences. External metabolic activation systems are required to test compounds affecting the genome via metabolites occurring in vivo. Animal-derived liver homogenate preparation containing phase I and II (S9 mix) are used to supplement test media to mimic the metabolic activation of the mammalian organism. Liver preparations from Aroclor 1254 or Phenobarbital/β-Naphtoflavone treated animals are required to obtain an active metabolism-simulating product. Thus, the idea of reducing animal testing by performing in vitro instead of in vivo tests is taken ad absurdum (Ku et al. 2007). Alternative animal-free approaches like the application of a biotechnological metabolisation system ewoS9R are implemented in this study.

The micronucleus test is recognised as one of the most important tools for assessing genotoxicity and is recommended by the OECD (487) for drug screening and ecotoxicological assessment (OECD Test Guideline 474 2014; OECD Test Guideline 487 2010; Hayashi 2016; Luzhna et al. 2013). Micronuclei (MNi) represent genetic damages, predicting somatic inherited and congenital genotoxin-transmitted impairments leading to malign transformation of cells (Kirsch-Volders et al. 2011). Two distinct approaches are basically employed in toxicological testing and environmental risk assessment, the in vivo micronucleus test (OECD Test Guideline 474 2014; Hayashi 2016) and the in vitro micronucleus test (OECD Test Guideline 487 2010; Luzhna et al. 2013).

MNi have been shown to arise either due to acentric chromosome fragments induced by clastogenic agents or due to spindle apparatus malfunctions caused by aneugens deterring the migration of whole chromosomes to the cell poles, with subsequent chromosome loss (Luzhna et al. 2013; Kirsch-Volders et al. 2002; Elhajouji et al. 2011; Fenech and Morley 1985; Fenech et al.^ 2011^).

It is postulated that cancer originates from alterations in the DNA of somatic cells exhibiting features of stemness (Greaves 2018). These alterations are propagated and result in augmented MNi frequencies for example in peripheral lymphocytes of patients with cancer or cancer-associated congenital syndromes compared to basal levels (Bonassi et al. 2007). Thus, highlighting the usefulness of MNi frequencies as a biomarker predicting cancer risks. The state of the art mammalian cell lines used for MNi-testing like Chinese hamster lung (V79), Human liver cancer (HepG2) or Chinese hamster ovary (CHO) cells are mainly tumour derived (OECD Test Guideline 487 2010). Therefore, the option to investigate the downstream fate of genotoxin-treated cells regarding hallmarks of carcinogenic transformation like the activation of oncogenes and the inactivation of tumour suppressor genes is not given.

Recent studies have shown that aneuploidy and multiple chromosome rearrangements are amongst the most common genetic features of cancer cells (Crasta et al. 2012; Forment et al. 2012). This phenomenon is denominated chromothripsis. Aneuploidy is linked to MNi formation and a correlation between aneugens, MNi formation and malign cell transformation has been suggested (Gordon et al. 2012; Giam and Rancati 2015). While the mechanisms leading to chromothripsis are still not completely elucidated, current evidence supports a model where chromosomes segregated into MNi are fragmented and subsequently reassembled and reincorporated into the main nucleus after mitosis (Crasta et al. 2012; Forment et al. 2012; Holland and Cleveland 2012; Korbel and Campbell 2013; Zhang et al. 2015). These scenarios suggest that MNi are directly involved in the process of carcinogenic transformation. It can be assumed that formerly “healthy” cells—in terms of proper cell cycle control—proven to be affected by genotoxins, bear a subpopulation of carcinogenic transformed cells. Therefore, MNi testing has forecasting power beyond the empirically based biomarkers and allows prediction regarding malign transformation (Crasta et al. 2012; Forment et al. 2012; Gordon et al. 2012; Giam and Rancati 2015; Holland and Cleveland 2012; Korbel and Campbell 2013; Zhang et al. 2015). In consequence, cancer research should strive for replacement of tumour-derived cells by “healthy” stem cells as in vitro model systems to study the process of carcinogenic transformation.

Concerning evaluation, MNi frequency assessment is traditionally performed by visual scoring of MNi in fixed and stained cells, a very time-consuming process unsuited for massive scale screening approaches. Nevertheless, automatic assessment of MNi frequencies has been validated for decades. One approach involves flow cytometry analyses of fluorescence-stained cells, and even protocols for discriminating between aneugenic and clastogenic mechanisms through differential staining have been described (Witt et al. 2008; Avlasevich et al. 2011; Di Bucchianico et al. 2017). These methods, however, require expert handling and, in the majority of the proposed techniques, the fixation or lysis of cells.

Alternatively, automated scoring of cells and MNi by digital image analysis has been proposed as a viable method for evaluating MNi induction even in living cells, with very promising results regarding reproducibility and reliability (Hayashi et al. 2007; Huang et al. 2011; Frieauff et al. 2013; Kanda et al. 1998). Particularly ingenuous is the application of systems relying on the constitutive expression of a histone-associated fluorescent protein signal for high-resolution imaging of chromosomes, which enables real-time investigation of chromosomal structures in living cells (Huang et al. 2011; Kanda et al. 1998). In this paper, results obtained with one such system, based on the constitutive expression of a histone2B-eGFP (H2B-eGFP) fusion protein in a transgenic stem cell line is presented.

Due to the lack of cell models showing features of “healthy stemness” in vitro, a stem cell line isolated from *Cyprinus carpio* brain has been established. This approach was triggered by the observation of persistent pluripotent cells in seasonal spawning fish. These cells are assumed to contribute to lifelong seasonal gonadal recrudescence and tissue regeneration being the driving factor for carp to have a more than 20-fold higher life expectancy than mammalian models like mouse and hamster (Levine 1997; Hurd and Ralph 1998; Tarín et al. 2005; Allner et al. 2010). Based on this observation, it was possible to isolate constitutive self-renewing cells from healthy individuals in a reproducible manner. The usage of a H2B-eGFP transgenic variant of this cell type to detect genotoxic effects will be reported in this paper. The dynamic H2B-eGFP signal architecture will be compared with the fixation and staining equivalents of MNi, nuclear buds and nucleoplasmic bridges which are used to assess genotoxicity in test procedures standardised thus far (Fenech 2007; Russo et al. 2019). To improve the impact of in vitro test in the context of replacement of animal experiments a biotechnological metabolisation system ewoS9R is implemented. Future perspectives in coupling MNi based non-destructive genotoxicity assessment with downstream monitoring of carcinogenic transformation of “healthy stem cells” in a single in vitro live imaging test procedure are discussed.

## Materials and methods

### Cell line and culture conditions

The KCB cell line has been derived from Carp (*Cyprinus carpio haematopterus*) brain primary cells (Stahlschmidt-Allner et al. in preparation). The cells have been modified to constitutively express a histone-2B-eGFP fusion protein (Kanda et al. 1998) to allow for chromosome/chromatin dynamics analysis. The latest *Mycoplasma* testing was done in June 2019.

The expression cassette of a CMV promoter-driven H2B-eGFP was derived of a H2B-eGFP plasmid (Kanda et al. 1998). H2B-eGFP was kindle provided by Geoff Wahl (Addgene plasmid # 11,680). The sequence is flanked by two repeats of the sea urchin arylsulfatase insulator (“Ars insulator”). The Ars insulator was placed in duplicate upstream and downstream of the coding sequence. The Ars insulator sequence was kindly provided by Masao Matsuoka (Hino et al. 2004; Tajima et al. 2006). The transgene sequence harbouring the expression cassette and the four copies of the Ars insulator are further flanked by piggybac terminal repeats. The sequences of piggybac terminal repeats were retrieved from pXL-BacII plasmid. pXL-BacII was kindly provided from Malcom Fraser (Cary et al. 1989). The sequence was assembled in silico and synthesised and subcloned by a commercial supplier (GeneArt Gene Synthesis by Thermo Fisher).

The transposon was introduced into wildtype KCB cells by co-transfection with a plasmid coding for the hyperactive piggyBac transposase (Yusa et al. 2011). Stably transfected cells were identified based on their eGFP fluorescence. To derive a clonal cell line, cells were dissociated into single cells and a limiting dilution was performed. Cells were plated on collagen-I coated multiwell plates to support clonal growth.

The genetically modified cell line has been deposited in accordance with the Budapest Treaty at the “Deutsche Sammlung von Mikroorganismen und Zellkulturen (DSMZ)” under the accession number DSM ACC3285. The cells are commercially available from GOBIO-GmbH, Aarbergen, Germany.

The self-signalling properties of nuclear structures allow for in situ observation of MNi by means of live imaging techniques. Repeated recording of size, position and shape of the nuclei is feasible.

### Cell culture

KCB cells grow adherent as a monolayer until confluence and then start to aggregate to dense clusters. Cells were pre-cultivated at 26 ± 1 °C under ambient CO_2_ conditions in sterile Cellstar® T25 tissue culture flasks (Carl Roth GmbH & Co. KG; CE48.1) using minimal essential medium (Gibco, Thermo Fisher Scientific Inc.; 21,575,097) supplemented with 10% foetal calf serum (Gibco, Thermo Fisher Scientific Inc.; 16,000,044) and 1% non-essential amino acids (Gibco, Thermo Fisher Scientific Inc.; 11,140,035), with 31.25 µg/mL Penicillin (Carl Roth GmbH & Co KG; CN28.1), 50 µg/mL Streptomycin (Carl Roth GmbH & Co. KG; 0236.1) and 50 µg/mL Amphotericin (Sigma Aldrich Corporation; A2942). Due to the poikilothermic nature of the carp, cells can be stored at 4–7 °C for several days up to weeks without recognisable impacts on vitality. This enables shipping of the cells as cell coated plates as components of ready to use test kits. The performance of the cell line according to the conditions above has been validated at GOBIO, Technische Universität Dresden and RWTH Aachen laboratories.

### Cell cycle length

To measure cell cycle length, DNA labelling of dividing cells according to the protocol of the manufacturer (Carl Roth GmbH + Co. HG; EdU Click-555; 7775.1) and time-lapse live-cell imaging of 40–60% confluent proliferating cultures at 26 °C were performed.

Live imaging-based cell counts of controls were conducted for three micronucleus experiments. Pictures were taken at the time points 0, 24 and 48 h with automatised live imaging instruments suited to take repeated pictures from the same area of a 96-well cell culture plate (Eppendorf AG; 0,030,730,119). The number of cells per picture was counted and the cell cycle length was calculated. Cell cycle data (interphase—cytokinesis duration) allocated to individual cells have been derived from time-lapse live imaging of cells cultured on 96-well cell culture plates recorded by IncuCyte (Sartorius) and Cytation 3 (Biotek Instruments).

### Micronucleus assay

For the evaluation of MNi frequencies, cells were cultivated at 26 °C in 96-well cell culture plates. Only the internal 60 wells were employed in tests to avoid evaporation effects in border wells called edge effect. After reaching a monolayer confluence of about 50 ± 10%, cells were exposed to the test substances diluted in the culture medium using DMSO (Carl Roth GmbH & Co. KG, A994.2) as a solvent. Solvent concentrations were kept at a maximum of 0.5% (v/v) in exposure media. Test compound and solvent-free exposure medium were also used as internal controls.

At least four different concentrations of each test substance and the internal control have been allocated to 12 wells each. Dosing was based on preliminary cytotoxicity range-finding tests. Exposure concentrations showing elevated cytotoxicity in definitive tests were not tested in all experiments and replaced in favour of lower concentrations. Tested substances included two aneugens [colchicine (Col; ≥ 94%, Carl Roth GmbH & Co. KG; 8884.1) and diethylstilbestrol (DES; ≥ 99% Sigma Aldrich Corporation; D4628)], two directly acting clastogens [4-nitroquinoline-1-oxide (4-NQO; ≥ 98% MP Biomedicals Inc., 155,926) and ethyl methanesulfonate (EMS; ≥ 99% Alfa Aesar; A12938)] and two substances that require metabolic activation before becoming clastogens [aflatoxin B1 (AFB1; Sigma Aldrich Corporation; 32,754) and cyclophosphamide (CP; Sigma Aldrich Corporation; 93,813)]. Cannabidiol (CBD; Sigma Aldrich Corporation; C6395) was included to investigate if the specific cytogenetic feature of nucleoplasmic bridges is displayed by this cell line as already known for human-derived cell cultures (Russo et al. 2019). Three different experimental designs for the micronucleus test with directly genotoxic substances were tested. The micronucleus frequencies were assessed after exposure to the compounds for 12 and 24 h or after exposure for 24 h with an additional 24 h of recovery. Additionally, the solvent control was tested after 24 h of exposure or 24 h of exposure and 24 h of recovery. Referring to the OECD guideline 487, the decision on the time point for MNi recording, to evaluate the suitability of the cell line, was related to cell cycle duration assumed to be ~ 24 h. For cannabidiol, the incidence of nucleoplasmic bridges has been recorded as an additional compound-specific parameter (Russo et al. 2019).

### Metabolic activation

The biotechnological alternative metabolic activation system, ewoS9R (EWOMIS), is based on a permanent rat liver cell line adopted to suspension cell culture and chemically defined medium without any animal-derived components such as foetal calf serum. The induction pattern has been optimised with various inducers to obtain a system with an especially pronounced CYP1A metabolisation capability evaluated by its conversion of 7-ethoxy-resorufin into resorufin. For ewoS9R production, enzyme-induced cells are harvested, homogenised and the S9 fraction is separated by centrifugation. The final concentration of the animal-derived S9 (Envigo) in the experiment was at 1.12 mg/ml and for the biotechnological alternative ewoS9R at 0.1 mg/ml. Further validation data on ewoS9R and KCB H2B-eGFP intrinsic metabolic capacities are given as online supplement (Supplementary Tables S1, S2 and Supplementary Figures S1, S2).

Experiments with compounds requiring metabolic activation were conducted at RWTH Aachen starting with ready to use 96-well cell-coated test-plates delivered from GOBIO lab. A pre-incubation approach was applied to allow metabolic activation at 37 °C according to the mammalian origin of the enzymes mixtures used. Rat liver S9 fraction and the biotechnological alternative ewoS9R were diluted in culture medium without FCS and test compounds were pre-incubated for 2 h at 37 °C and 150 rpm shaking, followed by sterile filtration with polyvinyl difluoride syringe membrane filters with 0.20 µm pores (Rotilabo®, Carl Roth GmbH & Co. KG; KC70.1) prior to cell exposure for 3 h at 26 °C.

Solvent control experiments with different DMSO concentrations were performed separately. Test runs have been performed for each test substance setup and for the solvent control tests. Referring to literature data (OECD Test Guideline 487, 2010) exposure periods for the clastogens that require metabolic activation (i.e. AFB1 and CP) were shorter than for directly genotoxic substances (3 h) but with an extended/additional recovery time (21 h or 45 h).

After exposure, pictures of cells were directly taken by an automated imaging system. Prior to the second read, the cells were allowed to recover for 24 h after replacement of the exposure medium by fresh culture medium.

### Image acquisition, analysis and MNi frequency evaluation

Three different imaging systems were used. 1. Pictures to present the eGFP signal architecture by fluorescence microscopy were taken from cells growing on cover glasses (Carl Roth GmbH & Co. KG; P233.1) in 12-well plates (Carl Roth GmbH; CE55.1), after fixation with paraformaldehyde (4% (v/v)); Carl Roth GmbH & Co.KG; 0335.2) and mounting on microscopic slides with Roti®Mount FlourCare (Carl Roth GmbH & Co.KG; HP19.1). Images were acquired with a Zeiss Axioscope 40 microscope (Carl Zeiss AG) using an external fluorescence light source (LEJ GmbH LQ-HXP 120 light source) and eGFP filterset (470/525; Carl Zeiss AG) in combination with a 2063 Full HD-HDMI-microscope camera (Di-Li).

2. For the first series of experiments (12 h exposure) live imaging microphotograps with 400-fold magnification were taken using an inverse microscope (Motic AE21; Motic Incorporation Ltd.) with an external fluorescence light source (LEJ GmbH LQ-HXP 120 light source) in combination with Endow GFP Bandpass Emission Filter (470/525; Motic Incorporation Ltd.) filters and an AxioCam MRc camera (Carl Zeiss AG). MNi were recorded visually and counted from microphotographs. Interphase nuclei counts were conducted using Image J software.

3. For automated live imaging, a Cytation 3 Cell Imaging Multi-Mode Reader (Biotek Instruments) was used. Two microphotographs (100-fold magnification) of each well in brightfield mode as well as an eGFP fluorescence recording were automatically acquired at each read. Total cell count was performed in GFP-channel pictures automatically using the Gen 5 software (v. 3.0) employing the built-in “cell count” protocol. Approximately 75% of the live images have sufficient quality enabling MNi quantification. From these pictures MNi were assessed visually. Only a MNi allocable to the main nucleus located in the same cell was considered in the MNi frequency evaluation. A guide for image analyses in terms of micronucleus identification, assessment of cytotoxicity and motility is provided as an online supplement (Supplementary Table S3 and Supplementary Fig. S3-S7). Additional time-lapse observations for about 72 h were conducted with Sartorius IncuCyte live imaging instruments.

### Statistical analysis

To assess the suitability of the cell line to recognise known carcinogens in compliance with standard testing protocols OECD, DIN, EN/ISO, the total number of nuclei and the related MNi frequencies exposed to the same concentration of the test compound in course of different experiments were pooled. In Fig. 3 an overview of the complete data set is given as box blots. Statistical analyses were based on the mean values of the combined data of the respective concentrations of the test compound in comparison to the control.

The Shapiro–Wilk test was applied to test normal distribution and the Brown Forsythe test was applied to test the equality of group variances. If these tests were passed, a two-tailed *t*-test was performed; otherwise, a Mann–Whitney Rank Sum test was performed. The statistical analyses were conducted with SigmaPlot V13 (Systat Software GmbH).

To assess dose-response relationships of the known genotoxins as a characteristic of the cell line, the regression coefficient of the equations representing the means of pooled experiments has been calculated and is given in Table 1. An overview of the data set derived from 1–4 independent exposure experiments using the same concentration of a test compound is given in Fig. 3.

**Table 1:** Shows The cumulated micronucleus frequencies of each concentration of the test compounds as well as the negative control after 24 h of treatment and 24 h of recovery (3 h of treatment and 45 h of recovery for clastogens that require metabolic activation), the total number of cells assessed and the statistical results including the *p*-values and functions

Compound	Concentration	Number of cells	Mean micronucleus frequency	*p*-value
4-Nitroquinoline *R*^2^ = 0.9811 *y* = 0.7151 + 18.6781x	Control	39,098	0.679	
	0.025 µM	7647	1.364	*Rp* = 0.003
	0.05 µM	24,734	1.708	*Rp* < 0.001
	0.1 µM	23,654	2.247	*R* p < 0.001
	0.2 µM	17,439	7.500	R *p* < 0.001
	0.3 µM*	2654	9.910	*Rp* = 0.002
Ethyl methanesulfonate *R*^2^ = 0.9313 *y* = 0.4378–0.1265x + 0.0485x^2	Control	12,905	0.415	
	1.28 mM	13,367	0.439	*Tp* = 0.409
	2.56 mM	15,157	0.320	*Tp* = 0.171
	3.84 mM	11,395	0.733	*Rp* = 0.016
	5.12 mM	8533	1.048	*Rp* < 0.001
Colchicine *R*^2^ = 0.9878 *y* = 0.7468 + 0.0028x + 0.0012x^2–1.3917E-005x^3	Control	29,834	0.778	
	0.031 µM	25,320	0.847	*Rp* = 0.004
	0.062 µM	23,577	1.464	*Rp* < 0.001
	0.125 µM	12,936	2.200	*Rp* < 0.001
	0.188 µM*	11,894	2.032	*Rp* < 0.001
Diethylstilbestrol *R*^2^ = 0.9923 *y* = 0.5098–0.0087*x* + 0.0003*x*^2 + 2.1490E-007*x*^3	Control	18,969	0.483	
	15 µM	7399	0.416	*Tp* = 0.887
	30 µM	15,238	0.746	*Rp* = 0.002
	45 µM	6310	0.636	*Tp* = 0.051
	60 µM	11,340	1.011	*Rp* < 0.001
	90 µM*	5796	2.469	*Rp* < 0.001
	120 µM*	2175	4.202	*Rp* < 0.001
Aflatoxin B1 + ewoS9R *R*^2^ = 0.9832 *y* = 0.5807 + 5.0574*x*− 2.017*x*^2	Control	3449	0.377	
	0.00625 mM	4030	0.596	*Tp* = 0.026
	0.0125 mM	4187	0.549	*Tp* = 0.116
	0.025 mM	3244	0.956	*Tp* = 0.001
	0.05 mM	2840	0.915	*Tp* = 0.012
	0.1 mM	3491	0.802	*Tp* = 0.002
	0.2 mM	2307	1.517	*Tp* < 0.001
	0.4 mM	2062	2.279	*Rp* = 0.004
	0.8 mM	1293	3.326	*Rp* = 0.004
	1.6 mM	793	3.531	*Rp* = 0.010
Aflatoxin B1 + S9 mix *R*^2^ = 0.984 *y* = 0.7125 + 5.0221*x*-1.9428*x*^2	Control	5550	0.631	
	0.00625 mM	4290	0.583	*Tp* = 0.503
	0.0125 mM	3495	1.059	*Tp* = 0.085
	0.025 mM	3346	0.837	*Rp* = 0.126
	0.05 mM	4395	0.865	*Tp* = 0.465
	0.1 mM	2673	1.085	*Rp* = 0.067
	0.2 mM	3263	1.502	*Tp* = 0.004
	0.4 mM	2237	2.414	*Rp* = 0.002
	0.8 mM	2281	3.595	*Rp* = 0.004
	1.6 mM	2367	3.718	*Rp* = 0.002
Cyclophosphamide + ewoS9R *R*^2^ = 0.8747 *y* = 0.5736 + 0.0067*x*− 1.1467E−005	Control	4046	0.420	
	1.132 µM	3702	0.594	*Tp* = 0.091
	2.266 µM	3644	0.302	*Rp* = 0.180
	4.531 µM	2570	0.817	*Rp* = 0.015
	9.063 µM	3307	0.605	*Rp* = 0.065
	18.125 µM	2340	0.812	*Rp* = 0.126
	36.25 µM	2871	1.219	*Rp* = 0.002
	72.5 µM	2156	1.855	*Rp* = 0.004
	145 µM	3477	1.179	*Rp* = 0.002
	290 µM	3008	1.496	*Rp* = 0.004
Cyclophosphamide + S9 mix *R*^2^ = 0.9922 y = 0.8913 + 0.0188x− 3.2630E-005	Control	2747	0.837	
	1.132 µM	3080	0.812	*Tp* = 0.711
	2.266 µM	2494	1.002	*Tp* = 0.590
	4.531 µM	2333	0.986	*Rp* = 0.937
	9.063 µM	2486	1.046	*Tp* = 0.667
	18.125 µM	2560	1.289	*Tp* = 0.018
	36.25 µM	2169	1.245	*Tp* = 0.073
	72.5 µM	2144	1.726	*Tp* = 0.017
	145 µM	2397	2.545	*Rp* = 0.002
	290 µM	2111	3.316	*Rp* = 0.002
DMSO *R*^2^ = 0.9907 *y* = 0.1637*x* + 0.4119	Control	15,961	0.407	
	0.25%	12,772	0.469	*Rp* = 0.912
	0.5%	13,704	0.492	*T* p = 0.159
	1.0%	10,067	0.559	*Tp* = 0.033
	2.0%*	8981	0.746	*Rp* = 0.013
Cannabidiol *R*^2^ = 0.8063 *y* = 0.0681*x* + 0.3034	Control	10,080	0.387	
	0.159 µM	11,512	0.487	*Tp* = 0186
	0.318 µM	10,423	0.441	*Rp* = 0.624
	1.590 µM	9392	0.532	*Tp* = 0.06
	3.180 µM	8857	0.700	*Rp* = 0.126

*Cytotoxicity threshold concentrations not tested repeatedly

## Results

**Fig. 1:**
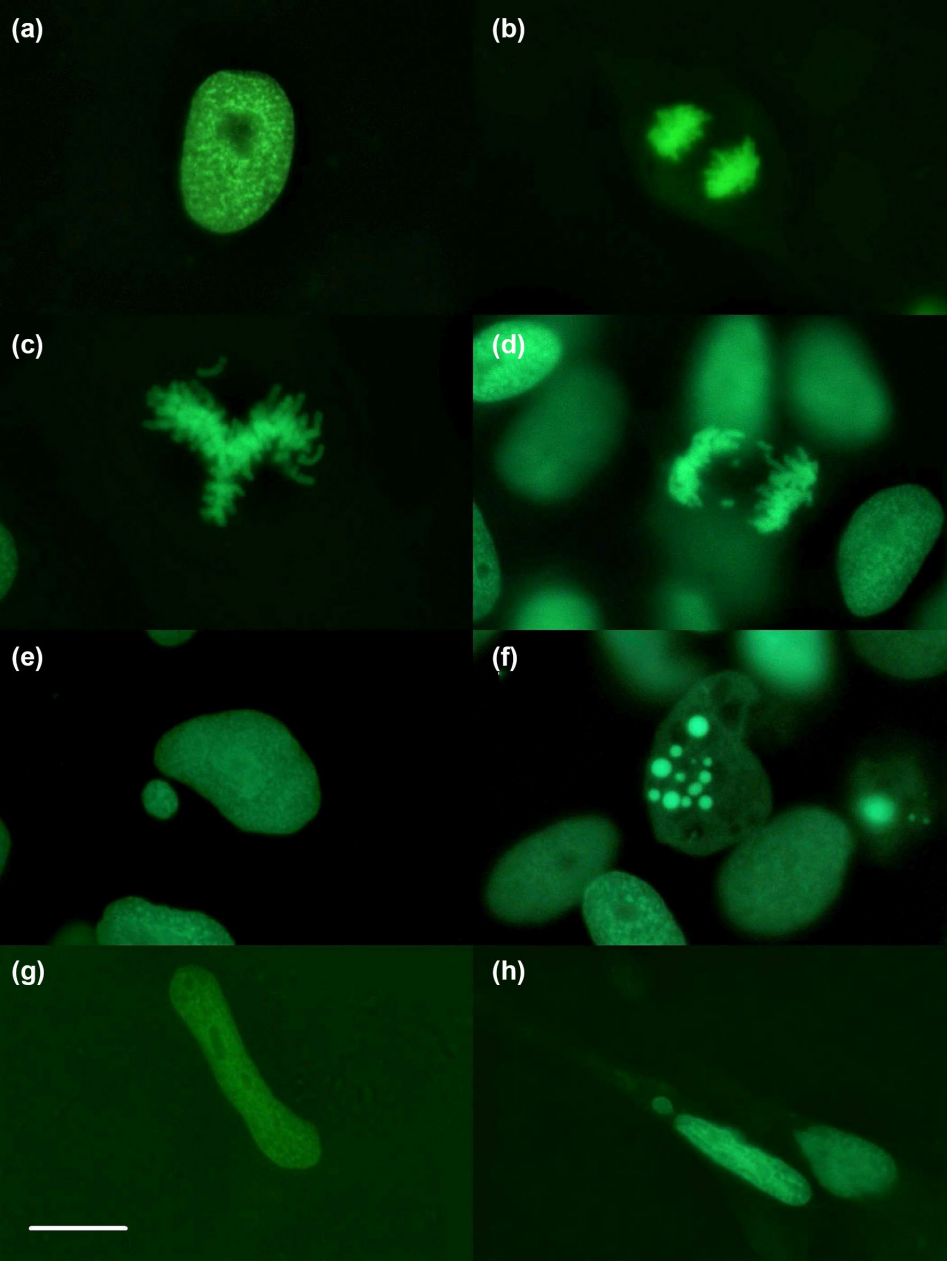
Nuclear structure of fixed cells with nuclei in interphase (**a**), during anaphase (**b**), during metaphase with difficulties to align properly (**c**), during anaphase with lagging chromosomes (**d**), as a main nucleus with a micronucleus beside (**e**), as a fragmented nucleus (**f**), as an elongated nucleus (**g**) and as a motile, elongated nucleus with a micronucleus beside (**h**). Pictures were taken in unexposed cultures (**a**, **b**), in colchicine exposed cultures (**c**, **d**), in 4-NQO exposed cultures (**e**, **f**) and in cannabidiol exposed cultures (**g**, **h**). Scale bar: 10 µm

**Fig. 2:**
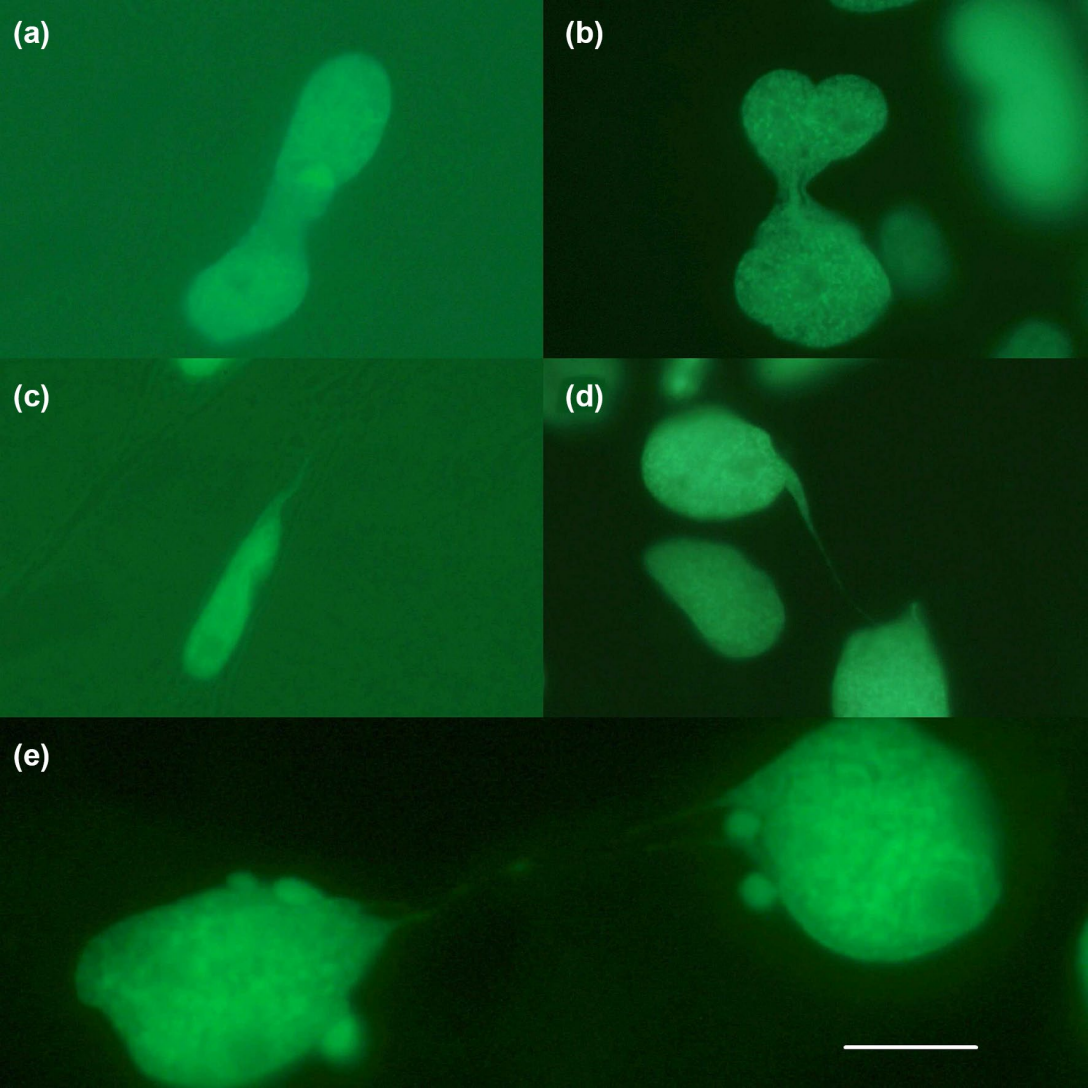
Nuclear structures of fixed cells after cannabidiol exposure. Solid nucleoplasmic bridges as a result of incomplete nuclear divisions (**a**, **b**). Filigree extrusion of nuclear material as a result of mechanical stress in the course of nuclear transport in cellular tubes (**c**). Filigree long-distance nucleoplasmic bridges of unknown genesis (**d**, **e**). Scale bar: 10 µm

Nuclear morphology, mitotic chromosome separation, induced and spontaneously occurring disturbances/alterations of nuclear morphology and chromosome segregation could be observed in fixed cells (Fig. 1) as well as in live imaging (Video (1). Due to the in situ mode of observation, it is possible to assess the health condition of each individual cell in terms of nuclear fragmentation of apoptotic cells versus cytogenetic damages in living cells (healthy cytoplasmatic condition) (Video (2). The nuclear eGFP signal widely corresponds to the results of conventional nuclear staining techniques like HE stain or the DNA RNA binding fluorescent dye acridine orange (Fenech et al. 2011). Solid H2B-eGFP labelled structures connecting two nuclei were recorded. These structures strongly correspond to nucleoplasmic bridges observed in human cells (Fenech et al. 2011). Additionally, a second type of filigree connections containing H2B-eGFP positive material between nuclei was detected (Fig. 2). The eGFP signal structure of filigree nucleoplasmic bridges appears during interphase and exhibits the corresponding non-condensed chromatin condition (Fig. 2). Thus, this structure differs from nucleoplasmic bridges in sensu stricto, thought to be related to dicentric (condensed) chromosomes (Forment et al. 2012; Holland and Cleveland 2012). This phenomenon was observed regularly in cannabidiol treated cells but neither in other treatments nor in controls.

Appearance and reintegration of MNi both also happening during interphase could be recorded (Video 3), thus contradicting the theoretical assumption that micronuclei mainly arise during mitosis. The process of cell movement is accompanied by squeezing of nuclei due to passaging through cell connecting tunnelling nanotube-like structures (Video 1). Cell proliferation features in terms of proportion and morphology of cells showing synchronous/asynchronous interphase/cytokinesis could be documented (Video 4).

### Cell cycle length

According to EdU staining of replicated DNA approximately, 69% of the KCB cells have undergone cell division after 24 h. Cell count based determinations of cell cycle lengths were conducted three times during a micronucleus test. The cell numbers were calculated prior to several experiments, immediately after exposure (24 h) and after an additional 24 h of recovery. The obtained percentages of untreated cells, which have undergone cell division after 24 h, were 58, 61 and 72% and 57, 68 and 70% after additional 24 h, respectively (Supplementary Table S4).

In view of the time-lapse results regarding the duration of individual cell division, this value represents the mean of two different cell populations not allocable to individual cells*.* Analyses of 38 time-lapse records indicate cytokinesis durations between 0.3 and 3.3 h. The interphase was observed to vary between 18.5–23.0 h for synchronous dividing cells and 28 h—> 33 h for asynchronous dividing cells (Supplementary Table S5). In summary, these data identify a heterogeneity of the KCB cells with respect to proliferation dynamics, where 30% of a culture show a prolonged interphase duration.

### Micronucleus assay

The testing capacity of a 96-well cell culture plate was found to be 4 different test concentrations of the test compound with 10 biological replicates in addition to solvent, positive and negative controls. Thus, for each test concentration even with reduced proliferation capacities 3000–4000 nuclei can be assessed.

### Micronucleus frequencies

To assess the suitability of the cell line to record MNi caused by known genotoxins, the results of at least two exposure experiments per substance except for AFB1, CP and CBD (only one experiment) were combined and the MNi frequencies in relation to the total number of examined cells of each tested concentration are given in Table 1. Exposure concentrations causing cytological features reflecting early stages of cytotoxicity, which are only recognisable with the applied self-signalling technologies, were not tested repeatedly. The complete dataset of each individual experiment is given in the supplement (Supplementary Tables S6–S13). Additional validation data in terms of intralaboratory and interlaboratory experiments as well as comparison of MNi frequencies with mammalian cell lines are given in the supplement (Supplementary Tables S14, S15, S16).

### DMSO

**Fig. 3:**
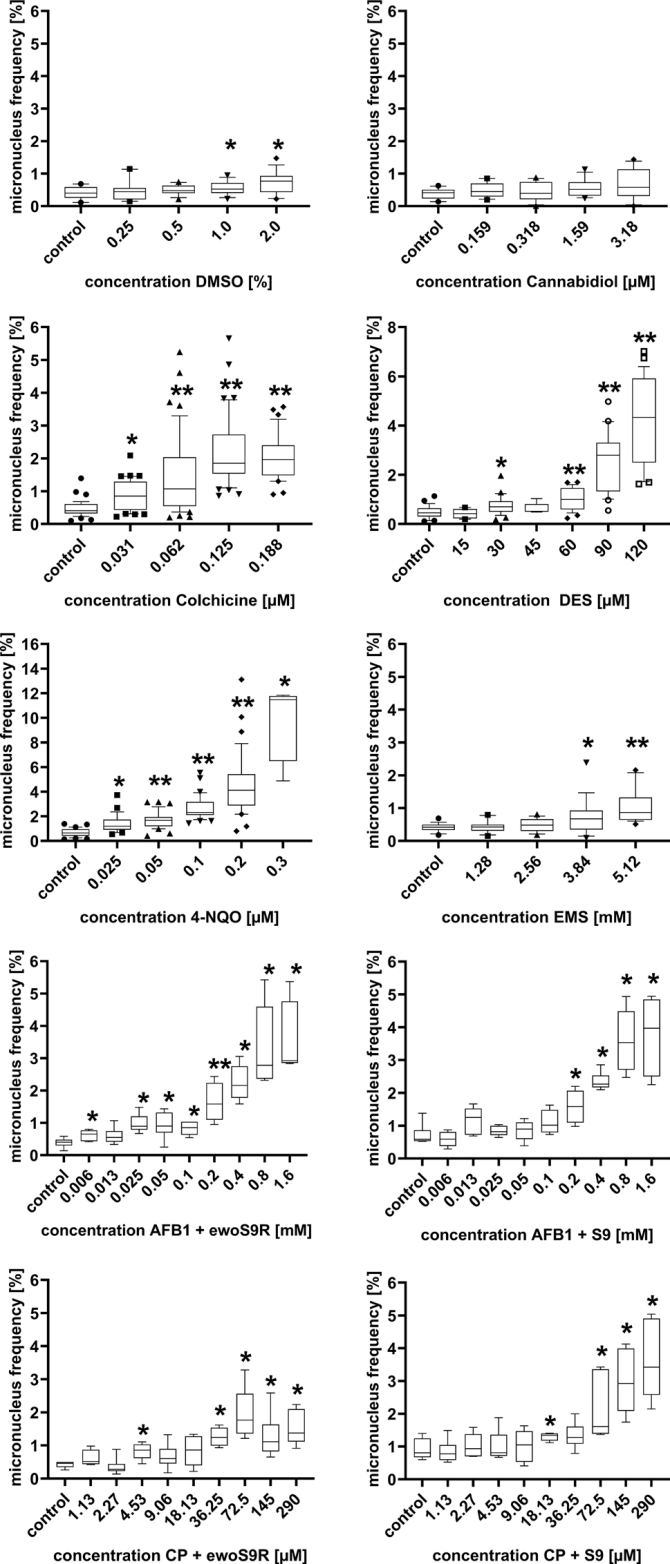
Pooled micronucleus frequencies of different wells/experiments of cells treated with different concentrations of DMSO, CBD, 4-NQO, EMS, Colchicine, DES, AFB1 and CP (AFB1 and CP both metabolic activated by animal S9 mix and a biotechnological ewoS9R enzyme mix) after 24 h treatment and additional 24 h recovery (3 h treatment and 45 h of recovery for AFB1 and CP) with the corresponding negative control for each compound. *p < 0.05 vs control; **p < 0.001 vs control

The micronucleus frequencies for DMSO, which was used as a solvent for many chemicals, are shown in Table 1 and Fig. 3. After 24 h of exposure and 24 h of recovery time, a statistically significant difference (*p* = 0.033; *p* = 0.013) between the control was only observed for the two highest concentrations (1.0 and 2.0%) of DMSO. Thus, the applied concentrations of maximal 0.5% are not suspected to increase micronucleus frequencies.

### Clastogens

The micronucleus frequencies were determined 24 h after removal of the test compound (Table 1). For all tested concentrations of 4-NQO (main positive control in standardised test protocols) a statistically significant difference between the micronucleus frequencies compared to unexposed cells was found. 4-NQO shows a linear dose-response relationship, which is a main validity criterion in genotoxicity testing. The two highest tested concentrations of EMS have a statistically significant difference compared with unexposed cells (*p* = 0.016; *p* =  < 0.001).

### Aneugens

The MNi frequencies increased statistically significant after exposure to colchicine in all concentrations compared to the control (Table 1 and Fig. 3).

The micronucleus frequencies of cells exposed to 30 µM and the three highest concentrations of diethylstilbestrol have a statistically significant difference compared with unexposed cells (*p* =  < 0.001).

### Cannabidiol

**Fig. 4:**
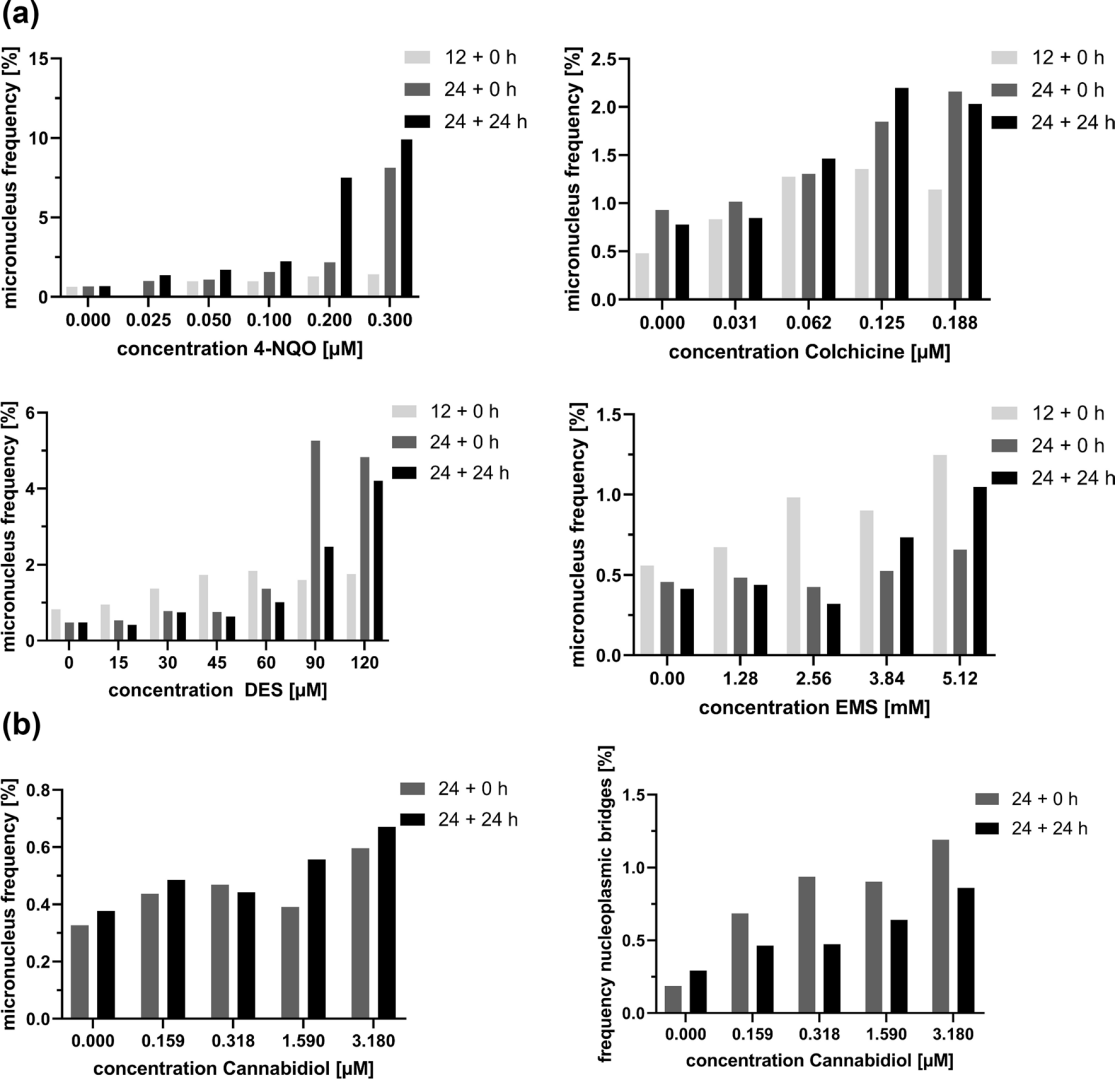
**a** Micronuclei induction by 4-NQO, colchicine, DES and EMS in KCB H2B-eGFP cells with varying exposure and recovery treatments. Micronucleus frequencies were assessed either after 12 h (12 + 0 h) or 24 h (24 + 0 h) of treatment and after 24 h of treatment with additional 24 h of recovery (24 + 24 h). **b** Solid nucleoplasmic bridges and MNi Frequencies observed at different time points of the same cannabidiol exposure experiment

Our observations suggest that micronuclei after exposure to cannabidiol arise from interphase nuclei in course of the translocation of nuclei (Fig. 1h). Thus, a mode of action in terms of aneugenic and clastogenic micronucleus formation cannot be allocated. There is only weak evidence for increasing MNi frequencies 24 h after exposure and additional 24 h of recovery, the mean assessment time points chosen for this study. A significant increase of MNi frequencies was observed directly after exposure to 3.18 µM of CBD without additional recovery time (Supplementary Table S13). Dose-dependent increasing incidences of solid nucleoplasmic bridges were observed at both investigated time points (Fig. 4 and Supplementary Table S17).

### Metabolic activation

The obtained micronucleus frequencies of all tested concentrations of AFB1 (Table 1) with both the animal derived and the biotechnological metabolisation system have a statistically significant difference compared with the unexposed cells (*P* =  < 0.001; except for AFB1 + rat S9 0.8 mM *P* = 0.004).

In addition, CP, a recommended positive control in international guidelines such as OECD or ISO was tested. The micronucleus frequencies of all tested CP concentrations obtained after exposure and metabolic activation with animal derived and the biotechnologically produced metabolisation system have a statistically significant difference (ewoS9R CP, *P* =  < 0.001; except for 18.125 mM with *P* = 0.011). The micronucleus frequencies of the cells exposed to different concentrations of CP with the addition of animal-derived S9 mix are statistically significantly different compared with the unexposed cells.

### Kinetic measurement of MNi frequencies

The comparison of MNi frequencies measured at different time points of an experiment are given in Fig. 3. The results of the 12 h exposure experiment are derived from one experiment whereas the results of 24 h exposure and no recovery time as well as 24 h exposure and additional 24 h recovery are derived from a distinct test series.

For colchicine (aneugen) and 4-NQO (clastogen) higher MNi frequencies were observed after prolonged exposure and recovery time (Fig. 4a). Statistically significant difference between the different MNi frequencies recorded at different time points of measurements and exposure regimes is given for individual exposure concentrations. However, the overall assessment of genotoxicity in compliance with toxicity criteria of OECD 487 indicates genotoxicity for all recording times, since at least one exposure concentration shows statistically significant elevated MNi frequency. This does not apply for cannabidiol where only the post-exposure MNi assessments indicate genotoxicity. Increased solid nucleoplasmic bridge frequencies are significant for all chosen assessment time points (Fig. 4b).

## Discussion

Our study shows the potential of H2B-eGFP transgenic, poikilothermic, non-tumour derived stem cells to broaden the perspectives of genotoxicity testing in combination with monitoring of malign cell transformation. The key advantage of the KCB H2B-eGFP cell line is the suitability for kinetic high throughput live imaging technology enabling the observation of the genesis of cytogenetic damages in addition to toxicological single endpoint measurements (OECD, ISO, DIN). Comprehensive insights into the genotoxic mode of action in terms of lost chromosomes, chromosomal breakage and motility-related mechanical stress are provided in a single approach (Fenech et al. 2011; Leslie 2016). The 4–7 °C storage option instead of cell-stressing cryopreservation enables the provision as ready to use 96-well cell-coated culture plate system.

There is no need for separate cytotoxicity/cell proliferation assessment, harvesting, hypotonisation, fixation, nuclear staining and counterstaining of dying cells. Thus, resulting in a reduction of work effort to less than 3.5 h for testing 4 concentrations of a test compound. The statistical soundness is improved since a considerable higher number of replicates and nuclei can be included in data analysis. A comparison of the workload with standardised protocols (OECD, DIN) is given as an online supplement (Supplementary Table S18). The workload can be reduced further using automatised evaluation and MNi assessment, making only a plausibility check necessary.

The condition of the cells can be evaluated by visual inspection of pictures or direct microscopy. In contrast to flow cytometry, the technique is fully accessible for plausibility checks of the automatised evaluation. Especially, the recognition of cytotoxic exposure concentrations and other impacts on the conditions of the cells is observable at a glance. The high throughput KCB H2B-eGFP technology enables easy exposure concentration range-finding and dose-response relationship assessment as a first step towards the mode of action prediction (Hashimoto et al. 2010). The performance of the tested cell line in definitive tests is directly comparable to those used in standardised test protocols. For the widespread used aneugen/clastogen (colchicine/4-NQO) positive controls, KCB cells proved to be more sensitive in terms of effective exposure concentrations (Antoccia et al. 1993; Valentin-Severin et al. 2003; Schmuck et al. 1988). A detailed comparison is given in supplementary Table 16. Especially 4-NQO, a compound causing DNA strand breaks via chemical interaction, caused the corresponding strongly dose-related increase in MNi frequencies (Fig. 3).

The low mean MNi frequency in the controls of the KCB H2B-eGFP cells of 0.58% reflects a stable mitosis control in contrast to immortalised cancer cells usually used for MNi testing that show higher basic MNi frequencies (Table 2). The in situ visualisation applied here, did not result in the induction of MNi frequencies exceeding 10%. Thus, we conclude that recordings of higher frequencies (Hashimoto et al. 2010) may be due to the uptake of extracellular nuclear material (Supplementary Fig. S6) (Bergsmedh et al. 2001), which cannot be allocated to dying neighbouring cells after trypsination and hypotonisation.

**Table 2:** Frequencies of spontaneously occurring micronuclei observed to date in different cell lines

Cell line	MNi frequencies (%)
KCB	0.58
V79 (Chinese hamster lung cell line) (Ellard and Parry 1993; Von der Hude et al. 2000)	0.86 or 0.9 ± 0.3*
CHL (Chinese hamster lung cell line) (Matsushima et al. 1999; Hashimoto et al. 2010; Kato et al. 2011)	0.91* or 1.6 or 0.70
HepG2 (Human liver cancer cell line) (Valentin-Severin et al. 2003)	1*
VH-16 (Primary human foreskin fibroblast) (Antoccia et al. 1993)	1.17 and 1.3*
HeLa (Human cervical cancer cell line) (Rao et al. 2008)	4.72
SHE (Syrian hamster embryo fibroblasts) (Schmuck et al. 1988)	0.72 ± 0.215

For the determination of MNi frequencies highlighted with (*), the cells were treated with cytochalasin B

The non-destructive automated quantification of MNi, the number of dividing vs dying cells and the recognition and quantification of binucleated cells/syncytial structures are short-term items to derive a more comprehensive dataset from MNi testing. This applies in particular to long-term time-lapse live imaging plate reads, enabling quantitative cell cycle analyses of individual cells.

Cannabidiol, known to induce nucleoplasmic bridges in addition to MNi in human tumour derived cells (Fenech 2007), elicited comparable effects in KCB. The observed filigree long-distance bridging nuclear structures are not described thus far. It remains to be elucidated if this is a peculiarity of the KCB specific non-tumour driven self-renewing pattern, or if the corresponding structures are not recognisable with standard procedures.

A crucial technical aspect of improving the forecasting power of cancer research in vitro is the compatibility of the approach to targeted metabolic activation techniques. The promising results of aflatoxin B1 and cyclophosphamide activation by a biotechnological non-animal derived S9 mix replacement opens beyond animal protection the following perspectives. Due to its biotechnological origin, its composition does not vary as it is produced according to biopharmaceutical principles. This approach eliminates variabilities that have been reported for the animal-derived products: External metabolic activation prior to exposure followed by sterile filtration enables separation of the activated test compounds from bacterial contaminants and cellular debris originating from the (enzyme) donor cells/tissue, thus, avoiding cellular bacterial infection and other responses to foreign cell compounds in downstream cultures of exposed cells and continuous exposure experiments. The technology is suited for spatial and timely targeted metabolic activation better reflecting the physiological conditions of different species or cancer susceptive tissues/organs or developmental events e.g. brain, kidney, placenta.

Kinetic data are only restricted available for the traditional in vitro MNi test methods, due to the high efforts in terms of replicate testing using destructive staining methods of MNi detection. A considerable increase in MNi frequency in the post-exposure period after 4-NQO treatment observed in this study has also been shown for immortalised mammalian V79 cells. Further, it could be confirmed for colchicine and 4-NQO that prolonged recovery time leads to higher MNi frequencies (Fig. 4a) as already shown by Pfeiffer et al. for V79 and for colchicine and vinblastine treatment by Jia et al. (Jia et al. 2016; Pfeiffer et al. 1998). For two test compounds DES and EMS it could be shown that MNi frequencies decrease (Fig. 4a).

In the light of the chromothripsis hypotheses, these findings call for kinetic imaging software tools to distinguish between the death of micronucleated cells and reintegration of MNi as a malign transformation- (chromothripsis-) triggering event.

In the case of cannabidiol the statistically significant increase of MNi frequencies was only observed directly after the exposure period. The effect seems to be reversible, indicating that MNi were either reintegrated or extruded. Concluding from these results, it is advisable to measure MNi frequencies at different post-exposure and post-recovery time points to obtain a better insight into the kinetic of MNi formation and thus, improving the forecasting power of in vitro genotoxicity testing.

The preliminary analyses of time-lapse recordings show considerable differences among individual KCB cells in terms of cytokinesis- and interphase duration. The assumption of a synchronous proliferation regime as known for tumour-derived cell lines does not apply for KCB cells. Long interphase resting cells and asynchronous cell division of daughter cells are known from stem cell niches in vivo (Hughes et al. 2012) and allow to attribute features of "healthy stemness" to KCB cells. Basing on the assumption that carcinogenesis rises from shifts in cell cycle control of slow dividing stem cells; a new set of cell cycle-related biomarkers can be implemented using KCB cells in cancer research (Greaves 2018).

The same applies to the assessment of motility displayed by elongated nuclei of KCB cells. Compounds inducing enhanced motility related mechanical stress can cause micronuclei (Supplementary Fig. S7) potentially followed by chromothripsis leading to malign transformation. Enhanced motility observed in downstream cultures is thought to reflect invasiveness, a hallmark of cancer. Thus, recording of elongated nuclei can supersede specific motility testing.

These new options to gather quantitative data regarding the health condition and fate of individual cells in situ will contribute to define the mode of action specific patterns of cytopathology and to forecast reversibility and hereditability of effects.

## Conclusion

The usage of constitutively H2B-eGFP expressing cells as screening tools for genotoxicity assessment has a high potential to replace bacterial systems like AMES and UMU chromo testing since it is possible to recognise a broader spectrum of the mode of actions including aneuploidy and mechanical stress-induced cytogenetic damages. The non-destructive evaluation of the cytogenetic status of the cell cultures enables kinetic measurements (downstream analyses) and continuous exposure regimes. The future perspective is to allocate alterations of gene expression profiles, cytological appearances, differences in responses to cell signalling as a consequence of exposure to carcinogens. Pluripotent, “healthy”, non-immortalised stem cells are a promising research tool to investigate carcinogenic cell transformation, starting with the initial steps towards the manifestation of cancer. In combination with sound non-animal derived biotechnological metabolic activation systems, the healthy stem cell approach presented here has a high potential to improve in vitro toxicology and to replace animal testing.

## Electronic supplementary material

Below is the link to the electronic supplementary material.

Supplementary file1 (DOCX 2312 kb)

Supplementary file2 (MP4 96619 kb)

Supplementary file3 (MP4 81403 kb)

Supplementary file4 (MP4 4248 kb)

Supplementary file5 (MP4 176379 kb)
