# Supplementary material for: Following the adverse outcome pathway from micronucleus to cancer using H2B-eGFP transgenic healthy stem cells
Source: Arch Toxicol. 2020 Jul 22;94(9):3265–80. doi: 10.1007/s00204-020-02821-3 (PMC7415759; doi:10.1007/s00204-020-02821-3)
Supplement: Supplementary file 1 — Supplementary file1 (DOCX 2312 kb) [file 204_2020_2821_MOESM1_ESM.docx]

Fig. S1: EROD activity of KCB cells and KCB cells/carp liver cocultures with and without exposure to the CYP450-inducing compound ß-naphthoflavone (<https://www.gobio-gmbh.de/wp-content/uploads/2020/05/VN_SB_GOBIO_PLUG-Druck.pdf>; <https://www.gobio-gmbh.de/wp-content/uploads/2020/05/S9-fraction-replacement.pdf>).

|  | Gene expression |
| --- | --- |
| Cyp19B | + |
| Cyp1A | + |
| Cyp1B | +* |
| Cyp1B2 | - |
| Cyp1C1 | +* |
| Cyp3A | + |
| GSTA | +* |
| MGST1 | +* |

Table S1: Gene expression of of metabolic enzymes in KCB H2B-eGFP cells (* inducible by known inductors) (<https://www.gobio-gmbh.de/wp-content/uploads/2020/05/S9-fraction-replacement.pdf>)

|  | UMU‑chromotest with ewoS9R (EWOMIS) | UMU-chromotest with rat derived S9 (Sekisui XenoTech, LCC) |
| --- | --- | --- |
| Growth Factor (positive control 2‑Aminoanthracene) | 0.96 ± 0.02 | 0.83 ± 0.02 |
| Induction Rate (positive control 2‑Aminoanthracene) | 4.35 ± 0.33 | 2.99 ± 0.27 |
| Growth Factor (solvent control DMSO) | 0.95 ± 0.04 | 0.95 ± 0.14 |
| Induction Rate (solvent control DMSO) | 0.94 ± 0.09 | 0.95 ± 0.11 |

Table S2: Effect-based characterisation of ewoS9R in the UMU-chromotest (DIN 38415-3:1996-12). Mean results of 3 UMU-chromotest including growth factor and induction rate after exposure to the solvent (DMSO) and the positive control (2-AA) with ewoS9R or with rat-derived S9

Fig. S2: Effect-based characterisation for endocrine potential of Benzo[a]pyrene after metabolisation via lyophilisated ewoS9R and rat liver-based S9. CALUX ® (chemically activated luciferase gene expression) assays using U2OS ERα CALUX ® cell line; x-axis logarithmic scale of B[a]P/nM concentration; y-axis ratio between sample relative luminescence units/control relative luminescence units. Error bars indicate SD


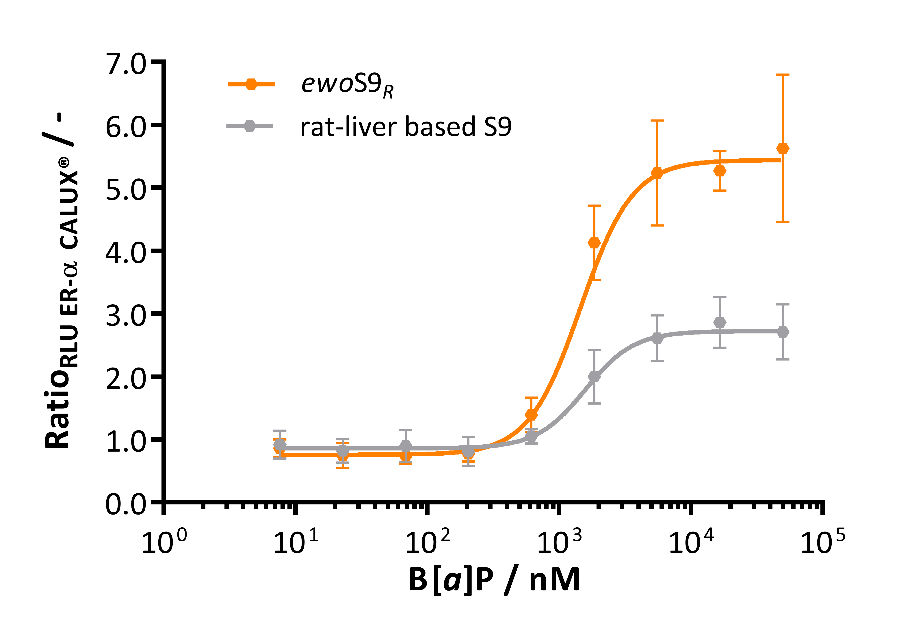


Table S3: Criteria for micronucleus counting, counting of fragmented nuclei as well as sessile and migrating nuclei

| micronuclei | fragmented nuclei |
| --- | --- |
| The size of a micronucleus should not exceed 1/3 of the main nucleus size | No main nucleus visible,  only nuclear fragments visible |
| Main and micronucleus have to be located in the same cell |  |
| The fluorescence intensity has to be at least as high as the fluorescence intensity of the main nucleus | The fluorescence intensity of nuclear fragments should be at least as high as the fluorescence intensity of main nuclei in the culture |
| Cells are attached to the surface of the well plate | Cells are detached from the surface of the well plate and have a roundish shape |
| Many micronuclei inside one cell have to be statistically counted as one micronucleus | Many nuclear fragments without a main nucleus in one cell will be statistically counted as one fragmented nucleus |
| Sessile nuclei | **Migrating nuclei** |
| Normal length-width ratio around 1 | Altered length-width ration |
| Cytoplasmic seam around the nucleus with enough space to the membrane, nucleus in the middle of the cell | Less or nearly none cytoplasmic seam around the nucleus; nucleus is located in a small extension of the cell |
| Weak light refraction of the cytoplasm | Strong light refraction of the cytoplasm |

Cell morphology of treated and untreated cells

Cytotoxic pattern of damage

Genotoxic pattern of damage

Untreated cells


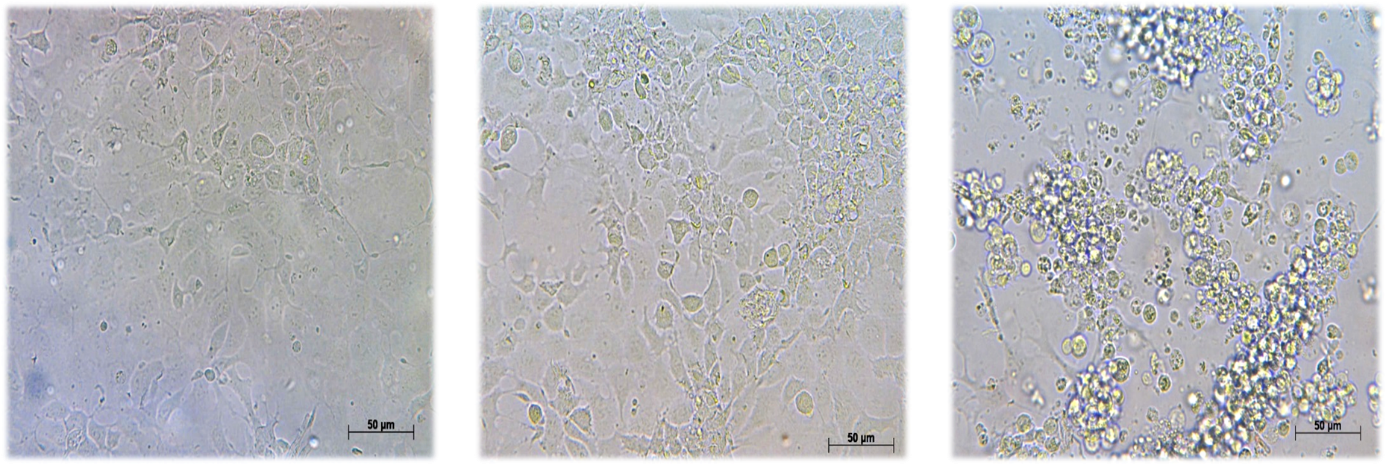


Fig. S3: Cell morphology of untreated and treated H2B-eGFP KCB cells to low and high concentrations of 4-NQO

Genotoxic pattern of damage

Dividing cell

Micronuclei

Dividing cell


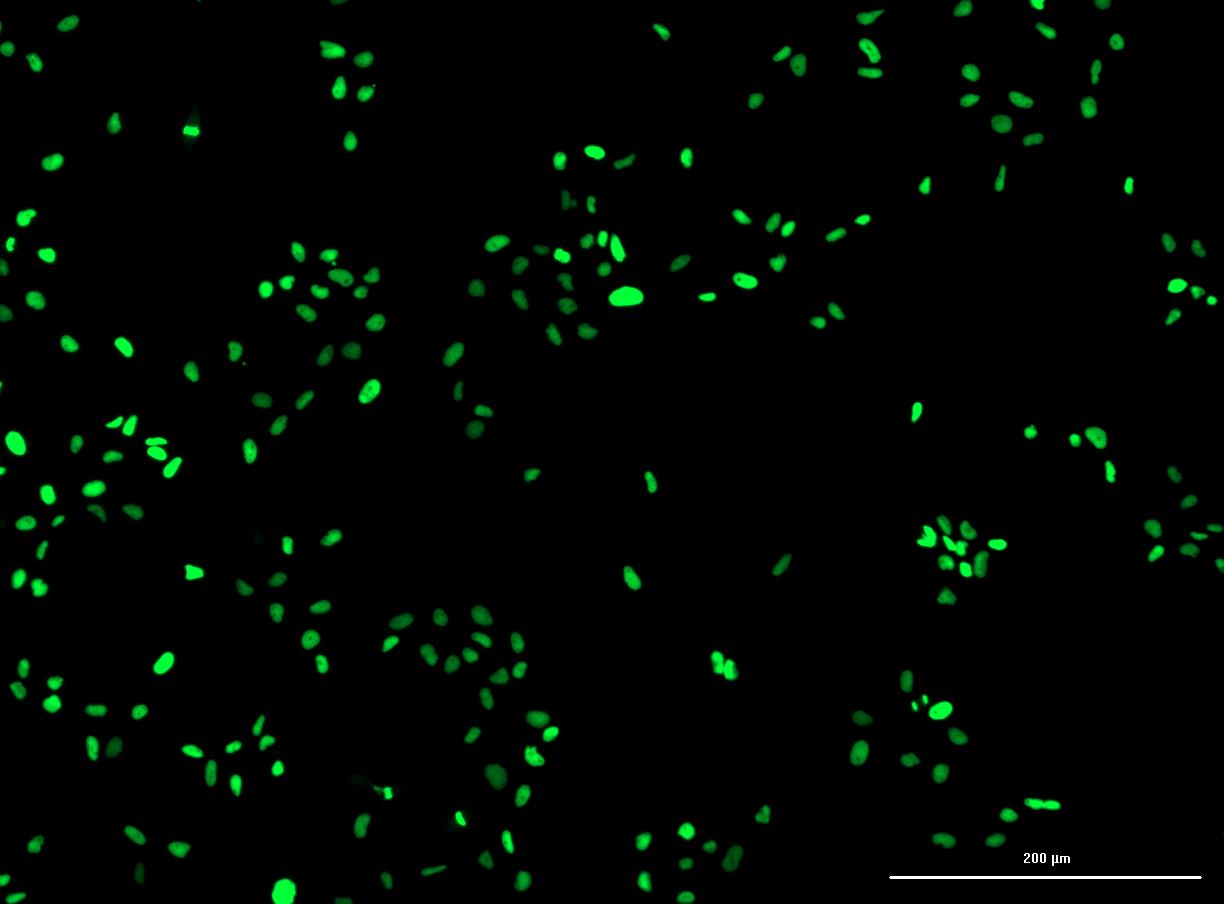
Fig. S4: Genotoxic pattern of damage with two additional dividing H2B-eGFP KCB cells after 4-NQO exposure

Result: 212 nuclei and 4 micronuclei

Micronuclei


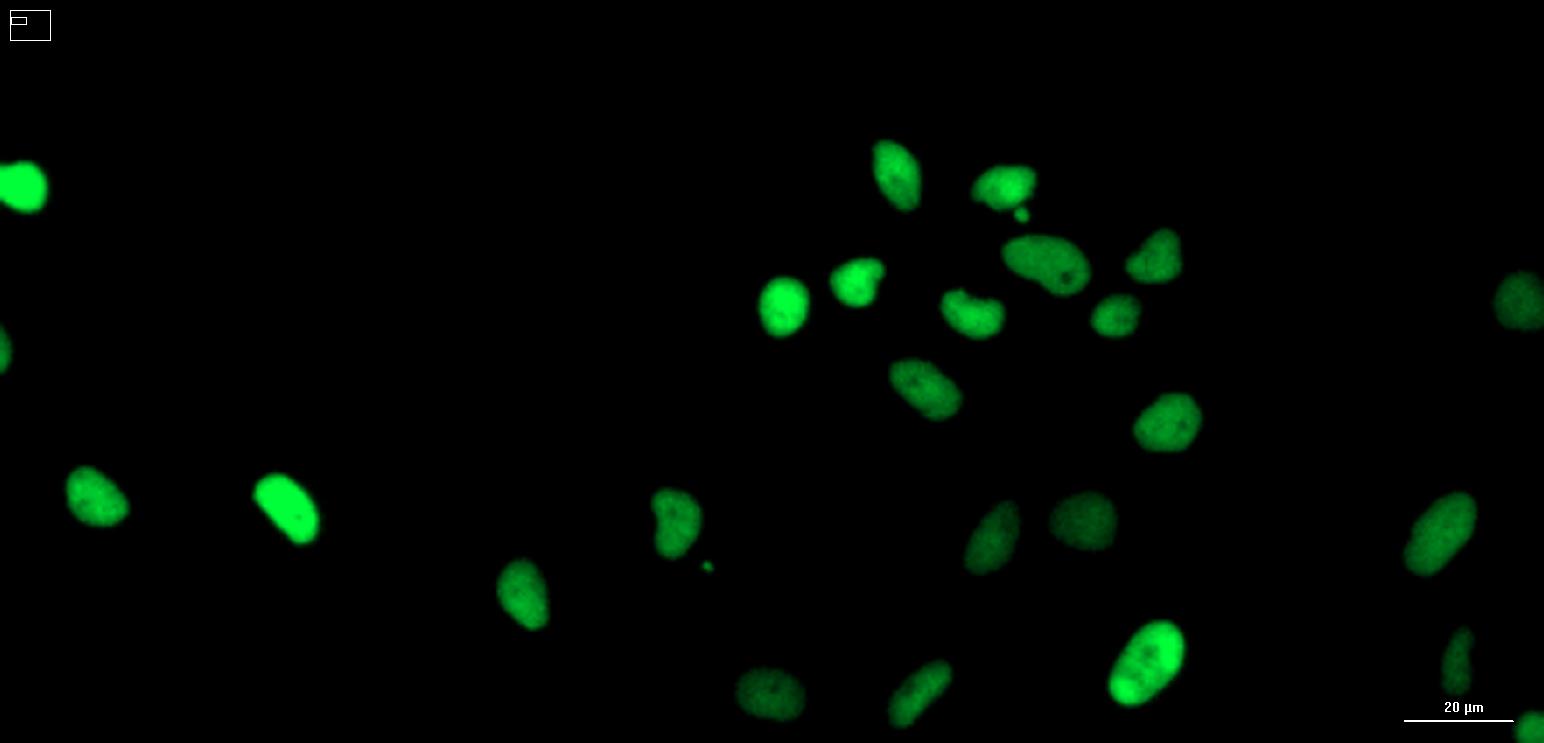


Micronuclei

Fig. S5: Enlargement of fig. S4 with the genotoxic pattern of damage of H2B-eGFP KCB cells after 4‑NQO exposure

Result: 21 nuclei and two micronuclei

Cytotoxic pattern of damage including fragmented nuclei


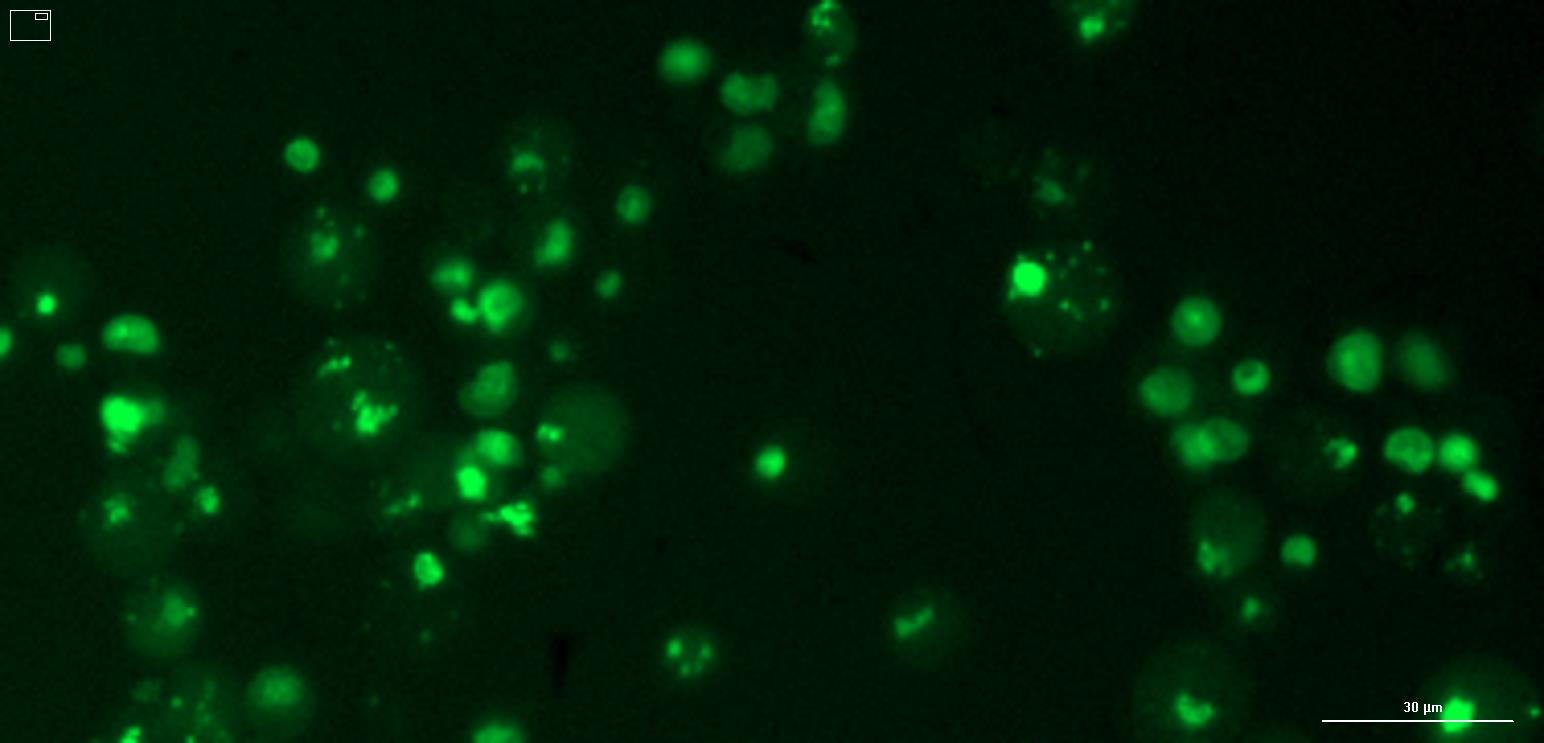

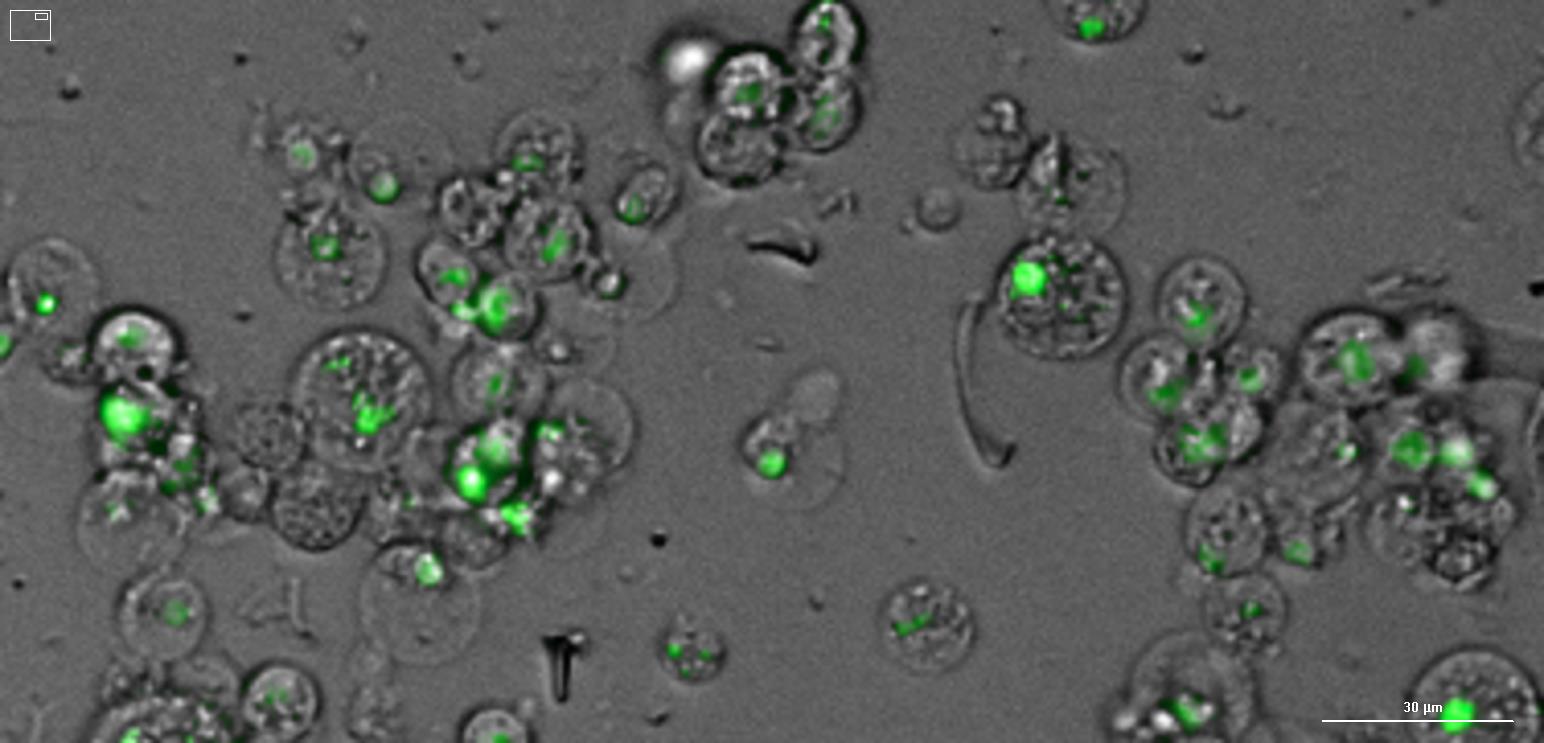


Fig. S6: Cytotoxic pattern of damage of H2B-eGFP KCB cells after exposure to a high concentration of 4-NQO including many roundish cell shapes with fragmented nuclei; (upper picture) only with GFP filter and (lower picture) with GFP filter and brightfield


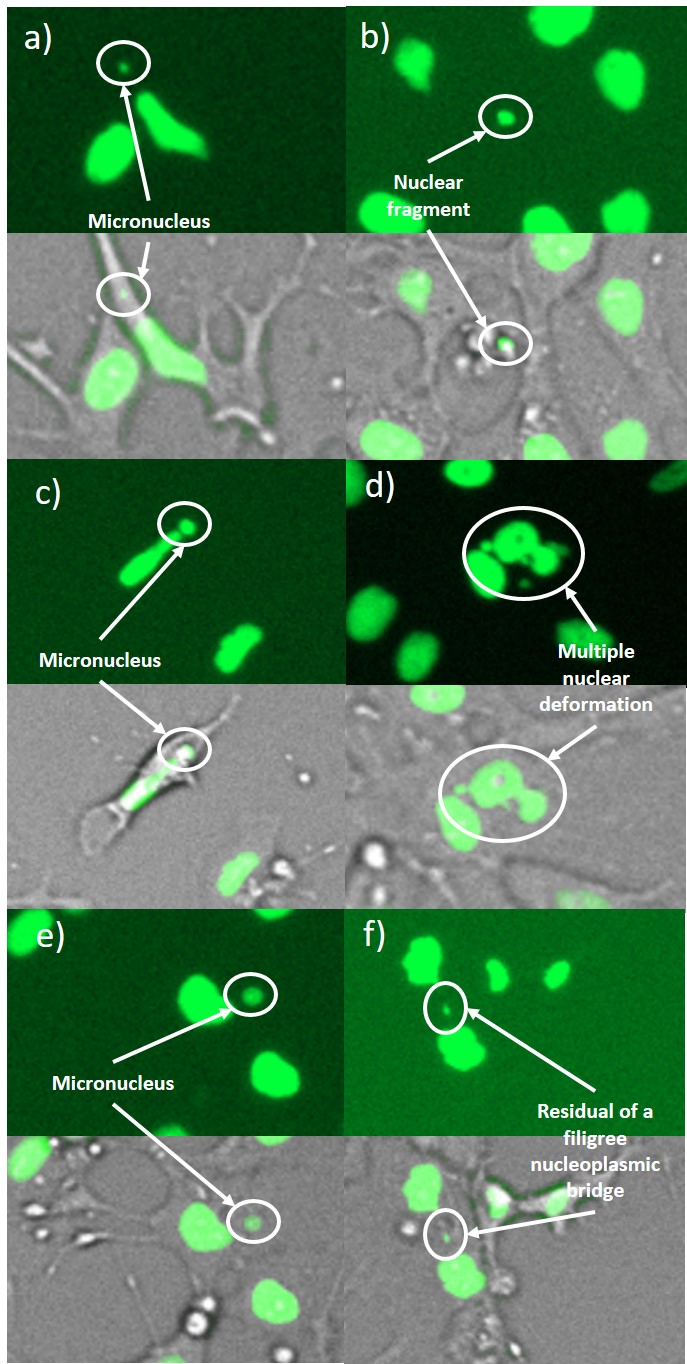


Fig. S7: Genotoxic pattern of damage with (a,b) micronuclei, (c) a large micronucleus, (d) a nuclear fragment outside of a cell, (e) a nucleus with multiple deformations and (f) a residual of a filigree nucleoplasmic bridge. Pictures were taken in 4-NQO exposed cultures (a,b,d), in colchicine exposed cultures (c,e) and in cannabidiol exposed cultures (f).

Table S4: Cell counts and the percentage increase in cell counts of untreated cells assessed at the time points 0, 24 and 48 h.

|  | 0 h | 24 h | 48 h |
| --- | --- | --- | --- |
| Experiment 1 | 10057 | 15839 (+58 %) | 26566 (+60 %) |
| Experiment 2 | 8113 | 13059 (+61 %) | 20499 (+57 %) |
| Experiment 3 | 7441 | 12786 (+72 %) | 21701 (+70 %) |

Table S5: Interphase duration between two mitoses of a cell and synchronously or asynchronously dividing daughter cells

| Interphase duration to the synchronous division of the daughter cells [h] | Interphase duration to the asynchronous division of the daughter cells [h] |
| --- | --- |
| 14.3 |  |
| 15.4 |  |
| 15.8 |  |
| 15.9 |  |
| 16.6 |  |
| 16.6 |  |
| 16.6 |  |
| 17.3 |  |
| 17.3 |  |
| 17.8 |  |
| 18.0 |  |
| 18.2 |  |
| 18.3 |  |
| 18.6 |  |
| 18.8 |  |
| 18.9 |  |
| 19.3 |  |
| 19.8 |  |
| 19.9 |  |
| 20.0 |  |
| 20.2 |  |
| 20.4 |  |
| 20.6 |  |
| 20.8 |  |
| 21.3 |  |
| 21.9 |  |
| 21.9 |  |
| 22.2 |  |
| 22.5 |  |
| 22.6 |  |
| 22.6 |  |
| 23.0 |  |
|  | 28.3 |
|  | 29.3 |
|  | 29.8 |
|  | 30.6 |
|  | 30.8 |
|  | 31.3 |

Table S6: Micronucleus frequencies assessed after 4-nitroquinoline treatment for 24 h or 24 h with additional 24 h of recovery (number of examined cells in parentheses)

| 4-NQO | control | 0.025 µM | 0.05 µM | 0.1 µM | 0.2 µM | 0.3 µM |
| --- | --- | --- | --- | --- | --- | --- |
| Experiment 1 (24 h exposure + 24 h recovery) | 0.139 % (720) | 1.124 % (445) | 3.150 % (254) | 2.247 (89) | 7.500 % (120) |  |
|  | 0.763 % (1179) | 0.957 % (627) | 1.699 % (412) | 2.545 % (393) | 4.118 % (170) |  |
|  | 0.267 % (749) | 1.109 % (451) | 1.954 % (614) | 2.017 % (347) | 2.821 % (390) |  |
|  | 0.417 % (1200) | 2.033 % (215) | 3.043 % (493) | 2.545 % (275) | 4.124 % (194) |  |
|  | 0.179 % (1120) | 3.721 % (448) | 1.863 % (322) | 2.222 % (225) | 7.031 % (128) |  |
|  | 0.885 % (1017) | 2.679 % (293) | 1.838 % (272) | 2.206 % (136) | 2.667 % (300) |  |
|  | 1.124 % (356) | 0.683 % (376) | 1.563 % (192) | 3.470 % (317) | 3.750 % (80) |  |
|  | 0.493 % (203) | 0.798 % (212) | 1.946 % (257) | 2.261 % (398) | 3.425 % (146) |  |
|  | 0.211 % (473) | 1.415 % (434) | 1.171 % (427) | 2.216 % (361) | 5.109 % (274) |  |
|  | 0.513 % (390) | 0.922 % (548) | 2.194 % (319) | 2.096 % (334) | 3.061 % (196) |  |
|  | 0.733 % (546) | 1.277 % (548) | 1.806 % (443) | 2.105 % (475) | 4.280 % (257) |  |
|  | 0.515 % (971) | 1.250 % (240) | 1.598 % (751) |  | 2.333 % (300) |  |
| Mean | 0.520 % | 1.497 % | 1.985 % | 2.357 % | 4.185 % |  |
| SD | ± 0.307 % | ± 0.895 % | ± 0.577 % | ± 0.405 % | ± 1.640 % |  |
| Significance | | R p < 0.001 | R p < 0.001 | R p < 0.001 | R p < 0.001 |  |
| Experiment 2 (24 h exposure + 0 h recovery) | 0.736 % (815) |  | 0.410 % (731) | 1.385 % (794) | 1.865 % (697) | 3.981 % (1281) |
|  | 0.490 % (817) |  | 0.338 % (592) | 0.508 % (787) | 1.199 % (1084) | 2.916 % (926) |
|  | 0.355 % (563) |  | 0.638 % (627) | 0.718 % (557) | 2.579 % (349) | 12.876 % (466) |
|  | 0.843 % (593) |  | 0.424 % (236) | 0.814 % (614) | 3.254 % (338) | 20.611 % (262) |
|  | 0.888 % (563) |  | 0.287 % (349) | 1.423 % (492) | 1.916 % (574) | 5.325 % (338) |
|  | 0.433 % (693) |  | 0.840 % (476) | 0.644 % (466) | 1.225 % (816) | 9.871 % (466) |
|  | 0.541 % (739) |  | 0.600 % (834) | 0.567 % (706) | 1.300 % (1000) | 6.787 % (1547) |
|  | 0.433 % (693) |  | 0.820 % (610) | 2.273 % (220) | 1.638 % (916) | 4.242 % (990) |
|  | 0.921 % (543) |  | 1.037 % (482) | 1.062 % (565) | 1.075 % (837) | 8.855 % (463) |
|  | 0.726 % (551) |  | 0.623 % (321) | 1.245 % (482) | 4.286 % (560) | 5.882 % (867) |
|  | 0.460 % (435) |  | 1.412 % (354) |  | 2.985 % (469) | 8.676 % (657) |
|  | 0.658 % (304) |  | 0.877 % (456) |  | 1.564 % (959) | 7.607 % (723) |
| Mean | 0.624 % |  | 0.692 % | 0.887 % | 2.074 % | 8.136 % |
| SD | ± 0.197 % |  | ± 0.326 % | ± 0.642 % | ± 0.999 % | ± 4.830 % |
| Significance | |  | T p = 0.540 | R p = 0.236 | R p < 0.001 | R p < 0.001 |
| Experiment 3 (24 h exposure+ 0 h recovery) | 0.708 % (424) | 1.446 % (415) | 1.429 % (280) | 1.878 % (213) | 3.488 % (172) |  |
|  | 0.469 % (426) | 0.815 % (368) | 1.271 % (236) | 4.000 % (150) | 3.659 % (82) |  |
|  | 0.538 % (372) | 1.278 % (313) | 2.232 % (224) | 2.186 % (183) | 1.600 % (125) |  |
|  | 0.847 % (236) | 0.662 % (302) | 0.901 % (222) | 1.484 % (337) | 2.312 % (173) |  |
|  | 1.014 % (296) | 0.917 % (327) | 1.195 % (251) | 3.086 % (162) | 1.351 % (74) |  |
|  | 0.737 % (543) | 0 % (305) | 0.654 % (153) | 2.564 % (117) | 2.963 % (135) |  |
|  | 0.570 % (526) | 0.971 % (412) | 2.198 % (364) | 1.058 % (189) | 0.952 % (105) |  |
|  | 0.726 % (413) | 1.449 % (276) | 1.091 % (275) | 1.511 % (397) |  |  |
|  | 0.602 % (332) | 1.931 % (259) | 1.488 % (336) | 2.516 % (159) |  |  |
|  | 1.072 % (373) | 0.800 % (250) | 1.754 % (285) | 2.283 % (219) |  |  |
|  | 0.623 % (321) | 0.743 % (269) | 2.941 % (238) | 1.916 % (261) |  |  |
|  | 0.461 % (217) | 0.905 % (221) | 0.806 % (124) | 2.469 % (162) |  |  |
| Mean | 0.697 % | 0.993 % | 1.497 % | 2.246 % | 2.33 % |  |
| SD | ± 0.198 % | ± 0.487 % | ± 0.677 % | ± 0.786 % | ± 1.072 |  |
| Significance | | T p = 0.064 | R p < 0.001 | R p < 0.001 | R p < 0.001 |  |
| Experiment 2 (24 h exposure+ 24 h recovery) | 0.972 % (2675) |  | 0.598 % (2341) | 2.014 % (2135) | 4.479 % (2054) | 4.870 % (1232) |
|  | 1.094 % (2559) |  | 1.268 % (1735) | 1.631 % (2207) | 4.440 % (1509) | 11.336 % (741) |
|  | 0.817 % (2080) |  | 1.887 % (1166) | 1.815 % (1267) | 7.369 % (977) | 11.833 % (431) |
|  | 1.128 % (1684) |  | 3.138 % (956) | 5.546 % (732) | 10.066 % (457) | 11.600 % (250) |
|  | 1.385 % (1588) |  | 1.901 % (789) | 3.872 % (1033) | 4.811 % (769) |  |
|  | 0.678 % (2211) |  | 1.221 % (1147) | 3.164 % (1201) | 4.408 % (1384) |  |
|  | 1.339 % (2465) |  | 1.636 % (2201) | 1.636 % (1589) | 2.171 % (1935) |  |
|  | 1.013 % (2467) |  | 0.994 % (1811) | 2.706 % (1517) | 5.094 % (1276) |  |
|  | 0.898 % (1447) |  | 1.184 % (1351) | 2.660 % (1579) | 5.987 % (1119) |  |
|  | 0.887 % (1692) |  | 2.268 % (882) | 3.986 % (577) | 13.111 % (389) |  |
|  | 0.627 % (1276) |  | 1.181 % (762) | 3.326 % (932) | 6.430 % (451) |  |
|  | 1.001 % (1199) |  | 1.478 % (1353) | 2.182 % (1650) | 8.869 % (902) |  |
| Mean | 0.987 % |  | 1.563 % | 2.878 % | 6.436 % | 3.303 % |
| SD | ± 0.231 % |  | ± 0.671 % | ± 1.174 % | ± 3.004 % | ± 3.366 % |
| Significance | |  | R p = 0.009 | R p < 0.001 | R p < 0.001 | R p = 0.004 |
| Experiment 3 (24 h exposure+ 24 h recovery) | 1.003 % (598) | 0.901 % (555) | 1.385 % (361) | 2.139 % (187) | 5.505 % (109) |  |
|  | 0.347 % (577) | 1.013 % (395) | 1.961 % (255) | 3.623 % (138) | 2.632 % (38) |  |
|  | 0.377 % (795) | 0.752 % (532) | 1.946 % (411) | 1.807 % (166) | 1.176 % (85) |  |
|  | 0.781 % (384) | 0.941 % (425) | 0.398 % (251) | 2.890 % (173) | 3.704 % (54) |  |
|  | 0.421 % (713) | 1.282 % (546) | 1.935 % (310) | 5.109 % (137) | 0.800 % (125) |  |
|  | 0.276 % (724) | 1.481 % (405) | 1.531 % (196) | 3.636 % (110) | 3.509 % (57) |  |
|  | 0.539 % (371) | 2.041 % (245) | 0.962 % (416) | 1.445 % (346) | 5.195 % (77) |  |
|  | 0.562 % (534) | 0.674 % (445) | 2.417 % (331) | 2.188 % (320) | 3.030 % (99) |  |
|  | 0.733 % (546) | 1.823 % (384) | 1.145 % (262) | 2.817 % (284) | 3.947 % (76) |  |
|  | 0.481 % (624) | 0.548 % (365) | 1.449 % (276) | 2.312 % (346) | 3.571 % (56) |  |
|  | 0.352 % (568) | 1.382 % (434) | 1.136 % (264) | 3.136 % (287) | 2.174 % (46) |  |
|  | 0.504 % (397) | 1.939 % (361) | 2.649 % (151) | 2.427 % (206) | 2.222 % (45) |  |
| Mean | 0.531 % | 1.232 % | 1.576 % | 2.794 % | 3.122 % |  |
| SD | ± 0.213 % | ± 0.508 % | ± 0.638 % | ± 0.993 % | ± 1.430 % |  |
| Significance | | R p < 0.001 | R p < 0.001 | R p < 0.001 | R p < 0.001 |  |

Table S7: Micronucleus frequencies assessed after ethyl methanesulfonate treatment for 24 h or 24 h with additional 24 h of recovery (number of examined cells in parentheses)

| EMS | control | 1.28 mM | 2.56 mM | 3.84 mM | 5.12 mM |
| --- | --- | --- | --- | --- | --- |
| Experiment 1  (24 h exposure + 0 h recovery) | 0.562% (356) | 0.572% (699) | 0.289% (692) | 0.471% (637) | 0.424% (472) |
|  | 0.488% (410) | 0.431% (464) | 0.361% (832) | 0.385% (519) | 0.430% (697) |
|  | 0.565% (531) | 0.513% (390) | 0.435% (690) | 0.152% (657) | 0.527% (569) |
|  | 0.463% (432) | 0.576% (694) | 0.470% (639) | 0.152% (657) | 0.792% (379) |
|  | 0.255% (783) | 0.290% (690) | 0.478% (836) | 0.415% (482) | 0.649% (462) |
|  | 0.420% (476) | 0.244% (410) | 0.287% (696) | 0.220% (911) | 0.454% (661) |
| Mean | 0.459 % | 0.438 % | 0.387 % | 0.299 % | 0.546 % |
| SD | ± 0.115 % | ± 0.143 % | ± 0.087 % | ± 0.141 % | ± 0.147 % |
| Significance | | T p = 0.783 | T p = 0.246 | T p = 0.057 | T p = 0.279 |
| Experiment 2  (24 h exposure + 0 h recovery) | 0.436% (459) | 0.212% (471) | 0.469% (640) | 0.545% (734) | 0.759% (527) |
|  | 0.280% (358) | 0.272% (734) | 0.182% (548) | 0.964% (415) | 1.064% (376) |
|  | 0.197% (508) | 0.489% (409) | 0.498% (402) | 0.480% (417) | 0.787% (508) |
|  | 1.124% (356) | 0.766% (653) | 0% (456) | 0.465% (645) | 0.475% (632) |
|  | 0.281% (356) | 0% (344) | 0.513% (585) | 0.394% (507) | 1.055% (474) |
|  | 0.326% (614) | 0.467% (214) | 0.370% (813) | 0.481% (624) | 0.853% (469) |
|  | 0.531% (377) | 0% (388) | 0.165% (605) | 0.390% (770) | 0.364% (549) |
|  | 0% (533) | 0.584% (685) | 0.830% (482) | 0.651% (307) | 1.064% (188) |
|  | 0% (359) | 0.661% (454) | 0.951% (526) | 1.408% (213) | 0.447% (447) |
|  | 0.469% (640) | 0.783% (511) | 0.145% (692) | 0.683% (439) | 0.375% (801) |
|  |  | 0.402% (497) | 0.375% (1066) | 0.675% (593) |  |
| Mean | 0.364 % | 0.422 % | 0.409 % | 0.649 % | 0.724 % |
| SD | ± 0.321 % | ± 0.276 % | ± 0.290 % | ± 0.302 % | ± 0.289 % |
| Significance | | T p = 0.665 | T p = 0.742 | R p = 0.015 | T p = 0.017 |
| Experiment 1  (24 h exposure + 24 h recovery) | 0.407% (492) | 0.149% (672) | 0.320% (937) | 0.813% (615) | 1.342% (298) |
|  | 0.380% (527) | 0.402% (746) | 0.680% (588) | 0.610% (492) | 0.784% (765) |
|  | 0.541% (739) | 0.367% (817) | 0.589% (679) | 0.890% (674) | 0.855% (351) |
|  | 0.465% (860) | 0.475% (842) | 0.469% (853) | 0.765% (654) | 0.642% (467) |
|  | 0.690% (435) | 0.759% (659) | 0.655% (611) | 0.681% (440) | 0.862% (464) |
|  | 0.416% (721) | 0.775% (774) | 0.752% (665) | 0.602% (665) | 0.866% (462) |
| Mean | 0.483 % | 0.488 % | 0.578 % | 0.727 % | 0.892 % |
| SD | ± 0.116 % | ± 0.242 % | ± 0.158 % | ± 0.116 % | ± 0.236 % |
| Significance | | T p = 0.966 | T p = 0.266 | T p = 0.005 | R p = 0.004 |
| Experiment 2  (24 h exposure + 24 h recovery) | 0.179% (1118) | 0.800% (377) | 0.313% (640) | 0.356% (1123) | 2.156% (371) |
|  | 0.344% (873) | 0.393% (764) | 0.215% (932) | 0.517% (773) | 1.166% (429) |
|  | 0.538% (372) | 0.444% (901) | 0.696% (862) | 0.153% (654) | 0.612% (654) |
|  | 0.225% (889) | 0.187% (1069) | 0.221% (907) | 0.669% (299) | 0.965% (311) |
|  | 0.378% (794) | 0.467% (1070) | 0.591% (508) | 2.392% (209) | 2.073% (193) |
|  | 0.437% (687) | 0.272% (367) | 0.361% (1384) | 0.094% (1069) | 1.847% (379) |
|  | 0.311% (964) | 0.415% (723) | 0.496% (806) | 1.027% (584) | 0.617% (648) |
|  | 0.530% (566) | 0.323% (928) | 0.804% (871) | 0.670% (896) | 1.316% (304) |
|  | 0% (447) | 0.444% (900) | 0.378% (794) | 1.358% (589) | 0.733% (546) |
|  | 0.319% (626) | 0.562% (890) | 0.181% (553) | 0.248% (404) | 0.638% (627) |
|  | 0.437% (686) | 0.424% (472) | 0.558% (1075) | 1.034% (290) | 0.509% (589) |
|  | 0.451% (1109) | 0.253% (396) | 0.268% (1492) | 0.311% (965) | 0.889% (675) |
| Mean | 0.346 % | 0.415 % | 0.423 % | 0.736 % | 1.127 % |
| SD | ± 0.155 % | ± 0.159 % | ± 0.204 % | ± 0.652 % | ± 0.595 % |
| Significance | | T p = 0.291 | T p = 0.304 | R p = 0.175 | R < 0.001 |

Table S8: Micronucleus frequencies assessed after colchicine treatment for 24 h or 24 h with additional 24 h of recovery (number of examined cells in parentheses)

| Colchicine | control | 0.031 µM | 0.062 µM | 0.125 µM | 0.188 µM |
| --- | --- | --- | --- | --- | --- |
| Experiment 1 (24 h exposure + 0 h recovery) | 0.785 % (1146) | 0.835 % (958) | 0.708 % (989) | 1.382 % (1592) | 2.004 % (948) |
|  | 0.928 % (1293) | 1.467 % (1500) | 1.320 % (1212) | 1.706 % (1055) | 2.210 % (724) |
|  | 0.952 % (945) | 1.715 % (1166) | 2.064 % (1599) | 3.020 % (1126) | 1.968 % (559) |
|  | 0.717 % (1255) | 1.120 % (1071) | 1.540 % (1169) | 2.459 % (854) | 1.729 % (347) |
|  | 0.801 % (749) | 1.132 % (795) | 2.515 % (835) | 1.293 % (928) | 2.419 % (744) |
|  | 1.110 % (1261) | 1.795 % (557) | 3.282 % (579) | 2.358 % (424) | 1.420 % (845) |
|  | 1.253 % (798) | 2.115 % (851) | 1.274 % (1256) | 1.178 % (1273) | 2.119 % (755) |
|  | 0.930 % (1290) | 1.126 % (1332) | 1.862 % (1128) | 2.130 % (751) | 2.038 % (638) |
|  | 0.929 % (1076) | 1.654 % (1209) | 0.901 % (999) | 2.093 % (1242) | 1.766 % (736) |
|  | 0.207 % (967) | 1.732 % (924) | 2.283 % (657) | 1.151 % (869) |  |
|  | 0.796 % (754) | 1.498 % (1068) |  | 1.786 % (728) |  |
|  | 0.451 % (443) | 1.600 % (875) |  | 1.849 % (703) |  |
| Mean | 0.822 % | 1.483 % | 1.775 % | 1.867 % | 1.964 % |
| SD | ± 0.278 % | ± 0.364 % | ± 0.788 % | ± 0.574 % | ± 0.293 % |
| Significance | | T p < 0.001 | R p = 0.004 | R p < 0.001 | T p < 0.001 |
| Experiment 2 (24 h exposure + 0 h recovery) | 0.703 % (569) | 2.989 % (435) | 3.901 % (282) | 4.032 % (124) | 3.226 % (124) |
|  | 1.639 % (366) | 2.618 % (955) | 1.534 % (326) | 1.575 % (127) | 7.018 % (171) |
|  | 4.800 % (125) | 1.513 % (793) | 3.204 % (437) | 3.358 % (268) | 4.348 % (322) |
|  | 1.258 % (318) | 0.550 % (1090) | 2.033 % (836) | 2.715 % (221) | 6.452 % (186) |
|  | 1.980 % (404) | 0.374 % (535) | 2.844 % (422) | 4.305 % (302) | 5.172 % (232) |
|  | 0.690 % (435) | 1.845 % (813) | 1.629 % (614) | 3.571 % (196) | 2.652 % (264) |
|  | 3.722 % (403) | 2.703 % (333) | 2.509 % (279) | 5.517 % (145) | 4.926 % (203) |
|  | 2.752 % (109) | 0.758 % (528) | 3.097 % (226) | 6.098 % (82) | 4.516 % (155) |
|  | 2.000 % (550) | 1.764 % (907) | 2.198 % (546) | 5.195 % (231) | 3.425 % (146) |
|  | 2.813 % (640) | 1.976 % (759) | 1.627 % (799) | 3.833 % (287) | 3.571 % (308) |
|  | 3.069 % (554) | 1.682 % (1011) | 1.179 % (424) | 3.043 % (230) | 2.475 % (202) |
|  | 1.873 % (694) | 1.485 % (808) | 1.280 % (547) | 2.482 % (282) | 3.429 % (350) |
| Mean | 2.275 % | 1.688 % | 2.253 % | 3.810 % | 4.267 % |
| SD | ± 1.220 % | ± 0.834 % | ± 0.863 % | ± 1.320 % | ± 1.429 % |
| Significance | | T p = 0.183 | T p = 0.960 | T p = 0.007 | T p = 0.001 |
| Experiment 3 (24 h exposure + 0 h recovery) | 0.568 % (352) | 0.821 % (487) | 0.871 % (459) | 0.813 % (246) | 1.460 % (274) |
|  | 0.911 % (439) | 1.114 % (449) | 0.283 % (353) | 0.880 % (341) | 1.511 % (331) |
|  | 0.411 % (487) | 1.018 % (491) | 1.705 % (352) | 1.266 % (316) | 0.877 % (228) |
|  | 0.417 % (480) | 0.270 % (371) | 0.857 % (350) | 0.678 % (295) | 1.684 % (297) |
|  | 0.412 % (729) | 0.318 % (314) | 0.692 % (289) | 0.858 % (233) | 1.984 % (252) |
|  | 0.235 % (425) | 0.809 % (371) | 1.613 % (310) | 0.737 % (407) | 1.119 % (268) |
|  | 0.388 % (515) | 0.242 % (413) | 0.833 % (360) | 1.905 % (315) | 1.873 % (267) |
|  | 0.654 % (306) | 1.114 % (359) | 0.552 % (362) | 1.320 % (303) | 2.000 % (250) |
|  | 0.758 % (396) | 0.744 % (403) | 0.494 % (405) | 2.090 % (335) | 1.288 % (233) |
|  | 0.855 % (351) | 0.893 % (448) | 0.512 % (391) | 0.830 % (241) | 0.881 % (227) |
|  | 0.445 % (449) | 0.249 % (402) | 0.932 % (429) | 1.379 % (290) | 2.066 % (242) |
|  | 0.637 % (314) | 0.265 % (377) | 1.294 % (309) |  | 3.571 % (196) |
| Mean | 0.558 % | 0.655 % | 0.887 % | 1.160 % | 1.693 % |
| SD | ± 0.208 % | ± 0.359 % | ± 0.445 % | ± 0.481 % | ± 0.725 % |
| Significance | | T p = 0.426 | T p = 0.030 | T p < 0.001 | R p < 0.001 |
| Experiment 4 (24 h exposure + 0 h recovery) | 0.230 % (868) | 0.664 % (602) | 0.852 % (352) | 1.761 % (284) | 0.990 % (101) |
|  | 0.409 % (733) | 1.370 % (146) | 0.658 % (456) | 0.962 % (312) | 1.156 % (173) |
|  | 0.179 % (560) | 0.388 % (258) | 0.380 % (263) | 2.069 % (145) | 2.604 % (192) |
|  | 0.414 % (724) | 0.426 % (235) | 0 % (259) | 1.439 % (139) | 1.887 % (212) |
|  | 0.190 % (526) | 1.186 % (506) | 0.637 % (157) | 1.250 % (400) | 1.220 % (82) |
|  | 0.503 % (596) | 0.561 % (535) | 0.402 % (498) | 1.190 % (336) | 0.901 % (222) |
|  | 0.540 % (556) | 0.395 % (253) | 0.765 % (392) | 1.852 % (216) | 2.674 % (187) |
|  | 0.233 % (429) | 0.472 % (424) | 0.725 % (414) | 1.005 % (199) | 1.079 % (278) |
|  | 0.563 % (355) | 0.452 % (442) | 0.408 % (245) | 1.124 % (89) | 3.315 % (181) |
|  | 0 % (248) | 0.906 % (331) | 2.367 % (169) | 0.521 % (192) | 1.603 % (312) |
|  | 0.923 % (325) | 0.348 % (287) |  |  | 0.347 % (288) |
| Mean | 0.380 % | 0.652 % | 0.719 % | 1.317 % | 1.616 % |
| SD | ± 0.252 % | ± 0.350 % | ± 0.630 % | ± 0.469 % | ± 0.907 % |
| Significance | | R p = 0.088 | R p = 0.139 | T p < 0.001 | R p < 0.001 |
| Experiment 5 (24 h exposure + 0 h recovery) | 0.285 % (701) | 0 % (320) | 0.644 % 621) | 1.022 % (587) | 0.735 % (272) |
|  | 0.222 % (450) | 0.140 % (716) | 0.627 % (319) | 0.662 % (453) | 0.333 % (300) |
|  | 0.433 % (693) | 0.267 % (375) | 0.473 % (423) | 1.093 % (366) | 1.695 % (413) |
|  | 0.302 % (663) | 0.828 % (483) | 0.429 % (466) | 1.319 % (379) | 0.604 % (497) |
|  | 0.829 % (362) | 1.247 % (481) | 0.896 % (335) | 0.325 % (308) | 0.769 % (390) |
|  | 0.631 % (317) | 0.824 % (364) | 1.215 % (247) | 0.850 % (353) | 0.909 % (330) |
|  | 0.429 % (466) | 0.597 % (670) | 1.379 % (580) | 0.771 % (519) | 0.525 % (381) |
|  | 0.288 % (694) | 0.293 % (341) | 0.528 % (379) | 0.474 % (422) | 1.014 % (493) |
|  | 0.329 % (607) | 0.499 % (401) | 0.606 % (495) | 0.597 % (335) | 0.939 % (639) |
|  | 0.353 % (567) | 0.592 % (507) | 0.474 % (422) | 1.577 % (317) |  |
|  | 0 % (409) | 0.244 % (410) | 1.279 % (391) | 0.631 % (317) |  |
|  |  | 0.307 % (326) | 0.627 % (319) |  |  |
| Mean | 0.373 % | 0.486 % | 0.765 % | 0.847 % | 0.836 % |
| SD | ± 0.216 % | ± 0.353 % | ± 0.341 % | ± 0.375 % | ± 0.388 % |
| Significance | | T p = 0.367 | R p = 0.003 | T p = 0.002 | T p = 0.003 |
| Experiment 2 (24 h exposure + 24 h recovery) | 1.395 % (215) | 1.395 % (430) | 3.604 % (222) | 0 % (195) | 0 % (213) |
|  | 0.905 % (221) | 1.121 % (1070) | 3.041 % (296) | 1,402 % (214) | 0 % (228) |
|  | 0.398 % (251) | 1.225 % (816) | 2.808 % (463) | 2.395 % (334) | 0 % (290) |
|  | 2.029 % (345) | 0.711 % (985) | 2.717 % (773) | 1.843 % (217) | 0 % (242) |
|  | 0.978 % (511) | 1.322 % (454) | 2.203 % (454) | 3.834 % (313) | 0 % (240) |
|  | 1.587 % (315) | 1.354 % (591) | 3.711 % (485) | 3.766 % (239) | 0 % (294) |
|  | 1.739 % (460) | 2.091 % (287) | 4.605 % (304) | 3.763 % (186) | 0 % (165) |
|  | 0 % (254) | 1.377 % (363) | 0.985 % (203) | 5.660 % (53) | 0 % (242) |
|  | 2.551 % (392) | 0.859 % (582) | 5.234 % (363) | 2.932 % (307) | 0 % (69) |
|  | 0.301 % (665) | 1.269 % (709) | 3.226 % (775) | 2.591 % (193) | 0 % (120) |
|  |  | 1.449 % (759) | 2.494 % (441) | 4.865 % (370) | 0 % (155) |
|  |  | 1.402 % (856) | 1.898 % (527) |  | 0 % (143) |
| Mean | 1.188 % | 1.298 % | 3.044 % | 3.005 % |  |
| SD | ± 0.818 % | ± 0.338 % | ± 1.158 % | ± 1.608 % |  |
| Significance | | R p = 0.895 | T p < 0.001 | R p = 0.007 |  |
| Experiment 3  (24 h exposure + 24 h recovery) | 0.438 % (685) | 0.508 % (788) | 1.835 % (436) | 1.119 % (268) | 2.715 % (221) |
|  | 0.614 % (814) | 0.954 % (524) | 1.597 % (501) | 1.581 % (253) | 1.818 % (275) |
|  | 0.483 % (828) | 1.402 % (428) | 1.563 % (384) | 1.736 % (288) | 3.093 % (194) |
|  | 0.682 % (733) | 1.117 % (537) | 1.581 % (506) | 1.984 % (252) | 2.521 % (238) |
|  | 0.286 % (1049) | 0.935 % (428) | 2.041 % (294) | 1.911 % (314) | 1.310 % (229) |
|  | 0.324 % (927) | 0.469 % (639) | 1.194 % (335) | 0.866 % (231) | 1.674 % (239) |
|  | 0.680 % (735) | 0.444 % (675) | 2.463 % (406) | 1.802 % (333) | 2.242 % (223) |
|  | 0.318 % (628) | 0.853 % (586) | 1.154 % (520) | 2.770 % (361) | 1.878 % (213) |
|  | 0.407 % (491) | 0.927 % (647) | 1.028 % (389) | 1.060 % (283) | 2.564 % (156) |
|  | 0.613 % (489) | 0.294 % (681) | 0.546 % (366) | 2.429 % (247) | 1.408 % (213) |
|  | 0.417 % (720) | 0.327 % (611) | 0.984 % (305) | 1.038 % (289) | 3.488 % (172) |
|  | 0.365 % (548) | 0.314 % (637) | 1.071 % (280) | 1.894 % (264) |  |
| Mean | 0.469 % | 0.712 % | 1.421 % | 1.683 % | 2.247 % |
| SD | ± 0.144 % | ± 0.365 % | ± 0.529 % | ± 0.583 % | ± 0.699 % |
| Significance | | R p = 0.126 | R p < 0.001 | R p < 0.001 | R p < 0.001 |
| Experiment 4  (24 h exposure + 24 h recovery) | 0.131 % (1529) | 0.870 % (230) | 0.248 % (807) | 1.598 % (438) | 1.364 % (220) |
|  | 0.331 % (1210) | 0.310 % (323) | 1.103 % (725) | 1.794 % (223) | 3.333 % (240) |
|  | 0.177 % (1132) | 1.015 % (394) | 1.495 % (535) | 1.863 % (161) | 2.4 % (250) |
|  | 0.371 % (1077) | 0.341 % (587) | 0.361 % (830) | 3.419 % (117) | 2.542 % (236) |
|  | 0.404 % (990) | 0.568 % (352) | 0.409 % (489) | 0.917 % (109) | 0.905 % (221) |
|  | 0.649 % (924) | 0.322 % (311) | 0.645 % (465) | 3.846 % (104) | 1.662 % (361) |
|  | 0.471 % (1061) | 0 % (196) | 0.442 % (906) | 1.794 % (446) | 1.961 % (357) |
|  | 0.404 % (990) | 0.223 % (449) | 0.368 % (815) | 2.386 % (461) | 2.339 % (342) |
|  | 0 % (228) | 0.408 % (735) | 1.236 % (647) | 1.149 % (348) | 2.025 % (395) |
|  | 0.229 % (437) | 0.677 % (443) | 0.5 % (400) | 1.623 % (308) | 2.010 % (398) |
|  | 0.493 % (406) | 1.471 % (272) | 1.015 % (394) | 3.111 % (225) | 2.128 % (376) |
|  | 0.299 % (335) | 1.047 % (191) | 0.588 % (170) | 1.653 % (242) | 1.839 % (435) |
| Mean | 0.330 % | 0.604 % | 0.701 % | 2.096 % | 2.042 % |
| SD | ± 0.177 % | ± 0.424 % | ± 0.407 % | ± 0.910 % | ± 0.610 % |
| Significance | | R p = 0.119 | T p = 0.008 | R p < 0.001 | R p < 0.001 |
| Experiment 5 (24 h exposure + 24 h recovery) | 0.269 % (1116) | 0.510 % (785) | 1.246 % (642) | 1.579 % (380) | 1.484 % (337) |
|  | 0.669 % (747) | 0.661 % (454) | 0.821 % (975) | 1.307 % (153) | 0.952 % (210) |
|  | 0.337 % (593) | 0.447 % (447) | 0.731 % (684) | 2.395 % (334) | 1.304 % (230) |
|  | 0.338 % (591) | 0.606 % (825) | 1.004 % (498) | 1.293 % (232) | 1.587 % (189) |
|  | 0.404 % (495) | 1.458 % (343) | 0.386 % (259) | 2.715 % (221) | 3.571 % (140) |
|  | 0.231 % (864) | 0.429 % (699) | 0.727 % (688) | 2.817 % (213) | 2.281 % (263) |
|  | 0.554 % (361) | 0.432 % (463) | 0.211 % (473) | 1.543 % (648) | 1.961 % (561) |
|  | 0.674 % (445) | 0.297 % (673) | 0.195 % (513) | 1.715 % (583) | 1.386 % (505) |
|  | 0.101 % (992) | 0.413 % (726) | 0.467 % (642) | 1.511 % (331) | 1.463 % (205) |
|  | 0.353 % (566) | 1.475 % (339) | 0.389 % (541) | 1.942 % (206) | 1.786 % (168) |
|  | 0.504 % (397) |  | 0.628 % (478) | 2.586 % (232) | 1.932 % (207) |
|  |  |  |  | 1.382 % (217) | 2.190 % (274) |
| Mean | 0.403 % | 0.673 % | 0.619 % | 1.899 % | 1.825 % |
| SD | ± 0.181 % | ± 0.430 % | ± 0.329 % | ± 0.574 % | ± 0.671 % |
| Significance | | R p = 0.098 | T p = 0.072 | R p < 0.001 | R p < 0.001 |

Table S9: Micronucleus frequencies assessed after diethylstilbestrol treatment for 24 h or 24 h with additional 24 h of recovery (number of examined cells in parentheses)

| DES | Control | 15 µM | 30 µM | 45 µM | 60 µM | 90 µM | 120 µM |
| --- | --- | --- | --- | --- | --- | --- | --- |
| Experiment 1  (24 h exposure + 0 h recovery) | 0.447% (447) |  | 1.166% (343) |  | 1.754% (228) | 5.952% (168) | 6.349% (126) |
|  | 0.703% (427) |  | 1.115% (269) |  | 0.939% (213) | 5.357% (168) | 1.515% (66) |
|  | 0.677% (443) |  | 0.889% (450) |  | 1,899% (316) | 4.878% (246) | 5.797% (138) |
|  | 0.855% (351) |  | 0.755% (265) |  | 1.173% (341) | 4.969% (161) | 3.268% (153) |
|  | 0.560% (357) |  | 0.883% (566) |  | 1.619% (556) | 4.026% (149) | 4.545% (88) |
|  | 1.053% (285 |  | 1.266% (237) |  | 1.488% (336) | 4% (200) | 4.762% (63) |
|  |  |  |  |  | 1.527% (131) | 8.065% (124) | 3.175% (126) |
|  |  |  |  |  |  | 5.056% (178) | 8.772% (114) |
|  |  |  |  |  |  | 6.25% (208) | 5.263% (152) |
|  |  |  |  |  |  | 7.051% (156) |  |
|  |  |  |  |  |  | 2.083% (144) |  |
|  |  |  |  |  |  | 5.455% (275) |  |
| Mean | 0.716 % |  | 1.012 % |  | 1.486 % | 5.262 % | 4.827 % |
| SD | ± 0.215 % |  | ± 0.198 % |  | ± 0.331 % | ± 1.539 % | ± 2.098 % |
| Significance | |  | T p = 0.032 |  | T p < 0.001 | R p < 0.001 | R p = 0.002 |
| Experiment 2  (24 h exposure + 0 h recovery) | 0.542% (738) |  | 0.549% (546) |  | 0% (83) | 0% (35) | 0% (3) |
|  | 0.171% (584) |  | 0.326% (307) |  | 2.041% (245) | 0% (33) | 0% (5) |
|  | 0.290% (345) |  | 0.641% (312) |  | 3.247% (154) | 0% (52) | 0% (99) |
|  | 0.332% (603) |  | 0.467% (428) |  | 1.220% (246) | 0% (58) | 0% (83) |
|  | 0% (729) |  | 0% (482) |  | 1.389% (72) | 0% (47) | 0% (89) |
|  | 0.282% (355) |  | 0.965% (311) |  | 1.190% (336) | 0% (75) | 0% (59) |
|  | 0% (289) |  | 1.538% (65) |  | 0% (77) | 0% (44) | 0% (37) |
|  | 0.615% (325) |  | 0.955% (419) |  | 1.377% (363) | 0% (49) | 0% (90) |
|  | 0% (135) |  | 0.882% (340) |  | 2.817% (142) | 0% (77) | 0% (28) |
|  | 0.322% (622) |  | 0.505% (396) |  | 0.847% (236) |  | 0% (30) |
|  |  |  | 1.123% (89) |  | 0.873% (229) |  | 0% (52) |
|  |  |  | 0.243% (412) |  |  |  | 0% (35) |
| Mean | 0.255 % |  | 0.683 % |  | 1.364 % |  |  |
| SD | ± 0.218 % |  | ± 0.427 % |  | ± 1.020 % |  |  |
| Significance | |  | T p = 0.01 |  | R p = 0.008 |  |  |
| Experiment 3 (24 h exposure + 0 h recovery) | 0.428 % (701) | 0.313 % (572) | 0.483 % (620) | 0.681 % (633) | 0.735 % (529) |  |  |
|  | 0.444 % (450) | 0.598 % (603) | 0.541 % (665) | 0.329 % (573) | 0 % (549) |  |  |
|  | 0.433 % (693) | 0.357 % (667 | 0.236 % (600) | 0.754 % (540) | 1.880 % (519) |  |  |
|  | 0.603 % (679) | 1.040 % (551) | 1.104 % (572) | 1.055 % (552) | 0.905 % (609) |  |  |
|  | 0.552 % (527) | 0.549 % (399) | 0.597 % (471) | 0.325 % (517) | 0.769 % (650) |  |  |
|  | 0.631 % (633) | 0.597 % (555) | 1.215 % (400) | 0.771 % (586) | 1.212 % (635) |  |  |
|  | 0.281 % (661) | 0.293 % (665) | 1.034 % (562) | 0.711 % (574) | 0.262 % (615) |  |  |
|  | 0.432 % (606) | 0.748 % (622) | 0.792 % (637) | 0.896 % (505) | 0.609 % (619) |  |  |
|  | 0.165 % (514) | 0.592 % (558) | 0.404 % (572) | 1.577 % (450) | 1.095 % (787) |  |  |
|  | 0.176 % (648) | 0.488 % (513) | 0.474 % (549) | 0.946 % (518) |  |  |  |
|  | 0 % (454) | 0.307 % (345) | 1.023 % (429) | 0.282 % (575) |  |  |  |
|  |  |  | 0.313 % (436) |  |  |  |  |
| Mean | 0.377 % | 0.535 % | 0.685 % | 0.757 % | 0.830 % |  |  |
| SD | ± 0.199 % | ± 0.225 % | ± 0.335 % | ± 0.377 % | ± 0.547 % |  |  |
| Significance | | T p = 0.097 | T p = 0.015 | T p = 0.008 | R p = 0.025 |  |  |
| Experiment 1 (24 h exposure + 24 h recovery) | 1.136 % (528) |  | 1.141 % (263) |  | 1.231 % (389) | 3.198 % (344) | 2.941 % (102) |
|  | 0.656 % (457) |  | 1.240 % (242) |  | 1.702 % (286) | 2.797 % (286) | 6.000 % (100) |
|  | 0.952 % (420) |  | 0.581 % (344) |  | 1.210 % (230) | 3.738 % (214) | 2.174 % (46) |
|  | 0.628 % (637) |  | 1.285 % (389) |  | 1.582 % (372) | 4.975 % (201) | 2.353 % (85) |
|  | 0.599 % (334) |  | 1.049 % (286) |  | 1.606 % (325) | 4.183 % (263) | 4.545 % (110) |
|  | 0.708 % (424) |  | 0.870 % (230) |  | 1.550 % (235) | 3.892 % (334) | 6.015 % (133) |
|  |  |  | 0.538 % (372) |  | 1.055 % (248) | 2.119 % (236) | 3.846 % (78) |
|  |  |  |  |  |  | 3.306 % (242) | 1.754 % (114) |
|  |  |  |  |  |  | 3.261 % (184) | 4.132 % (121) |
|  |  |  |  |  |  | 3.125 % (192) | 1.626 % (123) |
|  |  |  |  |  |  | 3.182 % (220) | 6.040 % (149) |
|  |  |  |  |  |  |  | 3.759 % (133) |
| Mean | 0.780 % |  | 0.958 % |  | 1.420 % | 3.434 % | 3.766 % |
| SD | ± 0.216 % |  | ± 0.304 % |  | ± 0.249 % | ± 0.751 % | ± 1.646 % |
| Significance | |  | T p = 0.258 |  | T p < 0.001 | T p < 0.001 | R p < 0.001 |
| Experiment 2 (24 h exposure + 24 h recovery) | 0.446 % (1122) |  | 0.847 % (236) |  | 1.307 % (153) | 1.064 % (94) | 4.494 % (89) |
|  | 0.190 % (1055) |  | 0.521 % (576) |  | 1.104 % (453) | 1.143 % (350) | 5.128 % (78) |
|  | 0.390 % (1026) |  | 0.702 % (570) |  | 1.608 % (311) | 0.552 % (181) | 4.494 % (89) |
|  | 0.315 % (634) |  | 0.850 % (353) |  | 1.004 % (498) | 4.124 % (97) | 6.742 % (89) |
|  | 0.139 % (720) |  | 0.353 % (283) |  | 0.758 % (132) | 1.508 % (199) | 6.061 % (66) |
|  | 0.114 % (874) |  | 0.939 % (639) |  | 0.565 % (177) | 1.455 % (275) | 4.615 % (65) |
|  | 0.305 % (655) |  | 0.253 % (792) |  | 0.922 % (434) | 2.857 % (140) | 5.660 % (53) |
|  | 0.145 % (691) |  | 0.494 % (809) |  | 0.656 % (305) | 1.342 % (298) | 1.695 % (118) |
|  | 0.457 % (656) |  | 0.999 % (801) |  | 1.375 % (291) | 1.576 % (571) | 7.018 % (57) |
|  | 0.510 % (392) |  | 0.407 % (737) |  | 1.685 % (178) | 1.084 % (369) | 4.167 % (72) |
|  | 0 % (271) |  | 1.961 % (153) |  | 0 % (100) | 0.976 % (205) | 3.448 % (58) |
|  | 0 % (505) |  | 0.829 % (362) |  | 1.104 % (453) | 1.329 % (301) | 2.128 % (47) |
| Mean | 0.251 % |  | 0.763 % |  | 1.007 % | 1.584 % | 4.638 % |
| SD | ± 0.177 % |  | ± 0.450 % |  | ± 0.474 % | ± 0.971 % | ± 1.653 % |
| Significance | |  | R p < 0.001 |  | R p < 0.001 | R p < 0.001 | R p < 0.001 |
| Experiment 3 (24 h exposure + 24 h recovery) | 0.556 % (1080) | 0.655 % (611) | 0.176 % (567) | 0.516 % (581) | 0.726 % (689) |  |  |
|  | 0.242 % (1241) | 0.196 % (511) | 0.500 % (1001) | 0.507 % (592) | 0.808 % (495) |  |  |
|  | 0.646 % (619) | 0.317 % (631) | 0.724 % (829) | 0.713 % (561) | 0.917 % (545) |  |  |
|  | 0.651 % (921) | 0.228 % (878) | 0.387 % (517) | 0.550 % (1091) | 0.598 % (502) |  |  |
|  | 0.609 % (821) | 0.440 % (909) | 0.445 % (449) | 1.038 % (578) | 0.233 % (430) |  |  |
|  | 0.375 % (533) | 0.198 % (505) | 0.588 % (680) | 0.489 % (613) | 0.565 % (354) |  |  |
|  | 0.455 % (880) | 0.610 % (492) | 0.593 % (675) | 0.918 % (1089) | 0.475 % (632) |  |  |
|  | 0.359 % (836) | 0.309 % (647) | 0.923 % (758) | 0.509 % (589) | 0.448 % (670) |  |  |
|  | 0 % (637) | 0.675 % (741) | 0.897 % (780) | 0.487 % (616) | 0.351 % (569) |  |  |
|  |  | 0.527 % (759) | 0.550 % (545) |  | 0.592 % (676) |  |  |
|  |  | 0.420 % (715) |  |  | 1.596 % (188) |  |  |
| Mean | 0.433 % | 0.416 % | 0.578 % | 0.636 % | 0.664 % |  |  |
| SD | ± 0.216 % | ± 0.181 % | ± 0.227 % | ± 0.208 % | ± 0.366 % |  |  |
| Significance | | T p = 0.852 | T p = 0.171 | T p = 0.058 | R p = 0.224 |  |  |

Table S10: Micronucleus frequencies assessed after aflatoxin B1 treatment with either rat-derived S9 or ewoS9R for 3 h and 21 h of recovery or 3 h and 45 h of recovery (number of examined cells in parentheses)

| AFB1 | 0 | 0.00625 mM | 0.0125 mM | 0.025 mM | 0.05 mM | 0.1 mM | 0.2 mM | 0.4 mM | 0.8 mM | 1.6 mM |
| --- | --- | --- | --- | --- | --- | --- | --- | --- | --- | --- |
| AFB1 + ewoS9R  (3 h exposure + 21 h recovery) | 0.888 % (563) | 0.469 % (639) | 0.371 % (539) | 0.913 % (438) | 1.248 % (641) | 1.247 % (481) | 1.481 % (540) | 1.354 % (443) | 3.672 % (354) | 2.408 % (706) |
|  | 0.230 % (868) | 0.376 % (532) | 0.652 % (460) | 0.709 % (988) | 0.931 % (537) | 0.366 % (547) | 1.391 % (575) | 2.094 % (382) | 4.893 % (327) | 3.086 % (324) |
|  | 0.444 % (900) | 0.941 % (425) | 0.563 % (710) | 1.141 % (789) | 2.041 % (392) | 1.443 % (485) | 1.705 % (352) | 3.488 % (344) | 2.946 % (577) | 3.5 % (200) |
|  | 1.124 % (445) | 1.235 % (405) | 0.503 % (995) | 0.840 % (595) | 0.547 % (731) | 1.978 % (455) | 0.767 % (391) | 1.429 % (560) | 7.463 % (268) | 7.143 % (14) |
|  | 0.294 % (681) | 0.808 % (743) | 0.419 % (955) | 0.833 % (600) | 0.865 % (578) | 1.772 % (395) | 1.878 % (426) | 1.995 % (401) | 4.986 % (361) | 5.882 % (34) |
|  | 0.475 % (842) | 0.381 % (787) | 0.612 % (327) | 0.797 % (878) | 0.656 % (762) | 1.237 % (485) | 0.809 % (371) | 2.778 % (432) | 6.944 % (360) | 4.337 % (415) |
| Mean | 0.576 % | 0.702 % | 0.520 % | 0.872 % | 1.048 % | 1.341 % | 1.339 % | 2.190 % | 5.151 % | 4.393 % |
| SD | ± 0.353 % | ± 0.351 % | ± 0.110 % | ± 0.147 % | ± 0.544 % | ± 0.561 % | ± 0.459 % | ± 0.819 % | ± 1.772 % | ± 1.802 % |
| Significance | | T p = 0.550 | T p = 0.719 | T p = 0.087 | T p = 0.105 | T p = 0.018 | T p = 0.009 | T p = 0.001 | R p = 0.002 | R p = 0.002 |
| AFB1 + R  (3 h exposure + 21 h recovery) | 1.325 % (604) | 1.224 % (490) | 0.980 % (510) | 2.717 % (368) | 2.471 % (607) | 1.818 % (605) | 1.938 % (516) | 2.610 % (498) | 3.171 % (473) | 2.834 % (494) |
|  | 0.629 % (636) | 0.680 % (735) | 2.374 % (674) | 1.212 % (825) | 2 % (450) | 1.832 % (546) | 2.181 % (550) | 1.599 % (563) | 2.148 % (512) | 2.194 % (547) |
|  | 0.971 % (309) | 2.989 % (435) | 1.412 % (708) | 2.033 % (541) | 2.056 % (535) | 1.399 % (429) | 2.664 % (563) | 2.765 % (434) | 1.995 % (401) | 1.979 % (657) |
|  | 0.687 % (873) | 0.576 % (694) | 0.984 % (915) | 1.235 % (405) | 1.162 % (516) | 1.412 % (708) | 2.326 % (473) | 3.141 % (382) | 2.717 % (368) | 2.207 % (589) |
|  | 0.896 % (1005) | 1.669 % (599) | 0.704 % (852) | 1.161 % (689) | 0.654 % (612) | 2.267 % (397) | 1.585 % (568) | 1.528 % (458) | 4.412 % (340) | 2.885 % (312) |
|  | 0.668 % (749) | 0.779 % (770) | 0.185 % (1626) | 1.014 % (888) | 1.165 % (601) | 1.698 % (530) | 3.351 % (415) | 3.351 % (388) | 2.895 % (380) | 2.412 % (539) |
| Mean | 0.862 | 1.320 | 1.107 | 1.562 | 1.585 | 1.738 | 1.983 | 2.499 | 2.890 | 2.418 |
| SD | ± 0.264 | ± 0.913 | ± 0.741 | ± 0.671 | ± 0.693 | ± 0.322 | ± 0.527 | ± 0.771 | ± 0.869 | ± 0.368 |
| Significance | | T p = 0.266 | T p = 0.465 | T p = 0.039 | R p = 0.093 | T p < 0.001 | T p < 0.001 | T p < 0.001 | T p < 0.001 | T p < 0.001 |

| AFB1 + ewoS9R  (3 h exposure + 45 h recovery) | 0.132 % (757) | 0.439 % (912) | 0.326 % (919) | 0.670 % (597) | 0.887 % (789) | 0.662 % (604) | 0.951 % (631) | 2.156 % (371) | 5.422 % (166) | 2.913 % (103) |
| --- | --- | --- | --- | --- | --- | --- | --- | --- | --- | --- |
|  | 0.587 % (681) | 0.687 % (728) | 0.617 % (810) | 0.946 % (740) | 1.282 % (390) | 0.543 % (737) | 1.520 % (921) | 2.446 % (368) | 2.778 % (252) | 2.830 % (212) |
|  | 0.412 % (485) | 0.803 % (498) | 0.446 % (896) | 0.830 % (482) | 0.851 % (470) | 0.775 % (645) | 1.643 % (426) | 3.057 % (458) | 2.315 % (216) | 5.366 % (205) |
|  | 0.354 % (565) | 0.416 % (721) | 0.478 % (627) | 1.108 % (361) | 0.248 % (403) | 0.929 % (538) | 2.432 % (329) | 1.587 % (252) | 3.761 % (452) | 2.930 % (273) |
|  | 0.442 % (452) | 0.755 % (530) | 0.642 % (467) | 0.846 % (591) | 1.433 % (349) | 1.033 % (484) |  | 1.958 % (613) | 2.415 % (207) |  |
|  | 0.393 % (509) | 0.624 % (641) | 1.068 % (468) | 1.480 % (473) | 0.911 % (439) | 1.035 % (483) |  |  |  |  |
| Mean | 0.387 | 0.621 | 0.597 | 0.980 | 0.935 | 0.830 | 1.636 | 2.241 | 3.338 | 3.510 |
| SD | ± 0.148 | ± 0.162 | ± 0.259 | ± 0284 | ± 0.412 | ± 0.203 | ± 0.610 | ± 0.553 | ± 1.297 | ± 1.238 |
| Significance | | T p = 0.026 | T p = 0.116 | T p = 0.001 | T p = 0.012 | T p = 0.002 | T p < 0.001 | R p = 0.004 | R p = 0.004 | R p = 0.01 |
| AFB1 + R  (3 h exposure + 45 h recovery) | 0.627 % (957) | 0.471 % (637) | 1.384 % (578) | 0.640 % (781) | 1.003 % (598) | 1.042 % (672) | 2.018 % (545) | 2.092 % (239) | 2.936 % (545) | 3.807 % (394) |
|  | 0.689 % (871) | 0.591 % (1015) | 1.252 % (559) | 0.962 % (520) | 1.217 % (822) | 1.629 % (614) | 1.525 % (590) | 2.853 % (631) | 4.039 % (619) | 2.584 % (503) |
|  | 1.377 % (363) | 0.874 % (801) | 1.661 % (542) | 0.786 % (636) | 1.063 % (847) | 0.993 % (705) | 0.980 % (510) | 2.422 % (289) | 2.472 % (364) | 2.251 % (311) |
|  | 0.523 % (1147) | 0.746 % (804) | 0.763 % (655) | 1.032 % (678) | 0.664 % (602) | 0.733 % (682) | 2.198 % (273) | 2.222 % (360) | 4.935 % (385) | 4.943 % (263) |
|  | 0.547 % (1280) | 0.290 % (1033) | 0.689 % (1161) | 0.821 % (731) | 0.795 % (755) |  | 1.636 % (550) | 2.315 % (216) | 3.533 % (368) | 4.130 % (460) |
|  | 0.536 % (932) |  |  |  | 0.389 % (771) |  | 1.132 % (795) | 2.191 % (502) |  | 4.817 % (436) |
| Mean | 0.717 | 0.595 | 1.150 | 0.848 | 0.855 | 1.099 | 1.582 | 2.349 | 3.583 | 3.755 |
| SD | ± 0.330 | ± 0.228 | ± 0.415 | ± 0.154 | ± 0.301 | ± 0.378 | ± 0.478 | ± 0.271 | ± 0.961 | ± 1.124 |
| Significance | | T p = 0.503 | T p = 0.085 | R p = 0.126 | T p = 0.465 | R p = 0.067 | T p = 0.004 | R p = 0.002 | R p = 0.004 | R p = 0.002 |

Table S11: Micronucleus frequencies assessed after cyclophosphamide treatment with either rat-derived S9 or ewoS9R for 3 h and 21 h of recovery or 3 h and 45 h of recovery (number of examined cells in parentheses)

| CP | control | 1.13 µM | 2.26 µM | 4.53 µM | 9.06 µM | 18.13 µM | 36.25 µM | 72.5 µM | 145 µM | 290µM |
| --- | --- | --- | --- | --- | --- | --- | --- | --- | --- | --- |
| CP + ewoS9R (3 h exposure + 21 h recovery) | 0.339 (590) | 0.559 % (537) | 0.334 % (599) | 1.129 (797) | 0.685 % (876) | 0.861 % (929) | 0.593 % (675) | 0.754 % (398) | 0.434 % (461) | 0.704 % (568) |
|  | 0.883 % (566) | 0.345 % (579) | 0.820 % (488) | 0.504 % (397) | 0.230 % (435) | 0.595 % (336) | 0.847 % (472) | 1.099 % (455) | 1.329 % (301) | 0.485 % (206) |
|  | 0.753 % (531) | 0.549 % (546) | 0.895 % (447) | 0.248 % (807) | 0.978 % (409) | 0.256 % (390) | 1.031 % (291) | 1.075 % (279) | 1.142 % (438) | 0.946 % (423) |
|  | 0.368 % (543) | 0.487 % (411) | 0.463 % (432) | 0.662 % (453) | 2.128 % (329) | 1.522 % (460) | 0.962 % (416) | 0.685 % (438) | 0.850 % (353) | 1.279 % (938) |
|  | 0.377 % (530) | 0.612 % (654) | 0.788 % (761) |  | 0.958 % (522) | 0.718 % (557) | 1.096 % (365) | 1.374 % (364) | 0.749 % (534) | 1.208 % (414) |
|  | 0.632 % (475) | 0.323 % (310) | 1.000 % (200) |  |  |  | 0.617 % (324) | 0.462 % (649) | 1.405 % (427) |  |
| Mean | 0.559 | 0.479 | 0.717 | 0.636 | 0.996 | 0.791 | 0.858 | 0.908 | 0.985 | 0.924 |
| SD | ± 0.231 | ± 0.119 | ± 0.260 | ± 0.371 | ± 0.701 | ± 0.466 | ± 0.212 | ± 0.333 | ± 0.373 | ± 0.335 |
| Significance | | T p = 0.469 | T p = 0.292 | T p = 0.693 | T p = 0.181 | T p = 0.309 | T p = 0.042 | T p = 0.061 | T p = 0.039 | T p = 0.061 |
| CP + S9 (3 h exposure + 21 h recovery) | 1.240 % (242) | 0.791 % (253) | 0.585 % (171) | 0 % (101) | 0 % (224) | 1.584 % (442) | 1.033 % (581) | 0.459 % (871) | 0.893 % (336) | 2.000 % (150) |
|  | 0.935 % (535) | 0.436 % (459) | 1.141 % (263) | 1.709 % (234) | 0.383 % (261) | 1.357 % (221) | 2.417 % (331) | 0.688 % (436) | 0.698 % (430) | 2.449 % (245) |
|  | 0.811 % (493) | 0.399 % (501) | 1.089 % (459) | 1.777 % (394) | 0.394 % (254) | 0.452 % (442) | 1.760 % (341) | 0.758 % (396) | 0.476 % (210) | 1.449 % (207) |
|  | 0.207 % (484) | 0.652 % (460) | 0.286 % (350) | 0.909 % (440) | 1.356 % (295) | 0.393 % (509) | 0 % (404) | 1.633 % (245) | 1.604 % (374) | 1.942 % (206) |
|  | 0.328 % (609) | 1.368 % (585) | 0.733 % (409) | 0.224 % (447) | 0.174 % (574) | 1.016 % (492) | 0.649 % (308) | 0.728 % (412) | 2.151 % (465) | 0.668 % (599) |
|  | 0.798 % (376) | 1.028 % (389) | 0 % (252) | 0.442 % (453) |  |  | 0.621 % (483) | 0.642 % (623) | 1.136 % (704) |  |
| Mean | 0.720 | 0.779 | 0.639 | 0.843 | 0.461 | 0.961 | 1.296 | 0.818 | 1.160 | 1.702 |
| SD | ± 0.387 | ± 0.371 | ± 0.447 | ± 0.759 | ± 0.526 | ± 0.531 | ± 0.777 | ± 0.413 | ± 0.622 | ± 0.678 |
| Significance | | T p = 0.792 | T p = 0.745 | T p = 0.730 | T p = 0.372 | T p = 0.406 | T p = 0.143 | T p = 0.680 | T p = 0.172 | T p = 0.014 |
| CP + ewoS9R (3 h exposure + 45 h recovery) | 0.260 % (768) | 0.421 % (949) | 0.255 % (1176) | 0.447 % (447) | 0.568 % (881) | 0.864 % (579) | 1.405 % (427) | 3.279 % (366) | 0.874 % (915) | 1.317 % (911) |
|  | 0.375 % (801) | 0.441 % (681) | 0.267 % (374) | 1.108 % (361) | 0.640 % (469) | 1.337 % (374) | 1.084 % (369) | 1.763 % (397) | 1.106 % (452) | 2.236 % (492) |
|  | 0.501 % (799) | 0.978 % (511) | 0.880 % (341) | 0.993 % (403) | 0.752 % (532) | 1.214 % (412) | 1.506 % (332) | 1.505 % (465) | 1.313 % (457) | 1.373 % (437) |
|  | 0.418 % (479) | 0.542 % (554) | 0.296 % (675) | 1.006 % (497) | 0.175 % (572) | 0.577 % (520) | 1.616 % (495) | 1.212 % (495) | 2.584 % (387) | 1.961 % (510) |
|  | 0.502 % (598) | 0.490 % (408) | 0.137 % (728) | 0.674 % (445) | 1.325 % (302) | 0.220 % (455) | 0.928 % (754) | 1.848 % (433) | 0.648 % (463) | 0.912 % (658) |
|  | 0.499 % (601) | 0.835 % (599) | 0.286 % (350) | 0.719 % (417) | 0.544 % (551) |  | 1.012 % (494) |  | 1.121 % (803) |  |
| Mean | 0.426 | 0.618 | 0.354 | 0.825 | 0.667 | 0.798 | 1.259 | 1.921 | 1.274 | 1.560 |
| SD | ± 0.097 | ± 0.232 | ± 0.264 | ± 0.252 | ± 0.376 | ± 0.537 | ± 0.287 | ± 0.798 | ± 0.681 | ± 0.532 |
| Significance | | T p = 0.091 | R p = 0.180 | R p = 0.015 | R p = 0.065 | R p = 0.126 | R p = 0.002 | R p = 0.004 | R p = 0.002 | R p= 0.004 |
| CP + S9 (3 h exposure + 45 h recovery) | 1.399 % (286) | 1.488 % (336) | 1.581 % (253) | 0.727 % (275) | 0.408 % (245) | 1.327 % (452) | 1.477 % (474) | 1.429 % (420) | 3.048 % (525) | 2.717 % (184) |
|  | 0.693 % (721) | 0.897 % (669) | 0.704 % (142) | 0.671 % (298) | 0.573 % (349) | 1.355 % (369) | 1.176 % (340) | 3.333 % (120) | 2.797 % (143) | 3.974 % (453) |
|  | 0.810 % (617) | 0.833 % (480) | 1.310 % (458) | 1.190 % (504) | 1.136 % (440) | 1.115 % (538) | 2.000 % (250) | 1.367 % (439) | 3.947 % (76) | 5.042 % (238) |
|  | 1.195 % (251) | 0.725 % (552) | 0.769 % (520) | 0.756 % (529) | 1.630 % (368) | 1.202 % (416) | 1.176 % (255) | 3.425 % (146) | 4.127 % (315) | 4.858 % (247) |
|  | 0.806 % (372) | 0.521 % (384) | 1.099 % (546) | 0.868 % (461) | 0.959 % (730) | 1.402 % (428) | 1.376 % (218) | 1.399 % (572) | 2.204 % (363) | 2.869 % (244) |
|  | 0.600 % (500) | 0.607 % (659) | 0.696 % (575) | 1.880 % (266) | 1.412 % (354) | 1.401 % (357) | 0.791 % (632) | 1.790 % (447) | 1.744 % (975) | 2.148 % (745) |
| Mean | 0.917 | 0.845 | 1.027 | 1.015 | 1.020 | 1.300 | 1.333 | 2.124 | 2.978 | 3.601 |
| SD | ± 0.311 | ± 0.344 | ± 0.367 | ± 0.462 | ± 0.473 | ± 0.117 | ± 0.403 | ± 0.985 | ± 0.940 | ± 1.202 |
| Significance | | T p = 0.711 | T p = 0.590 | R p = 0.937 | T p = 0.667 | T p = 0.018 | T p = 0.073 | T p = 0.017 | R p = 0.002 | R p = 0.002 |

Table S12: Micronucleus frequencies assessed after DMSO treatment for 24 h or 24 h with additional 24 h of recovery (number of examined cells in parentheses)

| DMSO | control | 0.25 % | 0.5 % | 1 % | 2 % |
| --- | --- | --- | --- | --- | --- |
| Experiment 1  (24 h exposure + 24 h recovery) | 0.394 % (1016) | 0.149 % (670) | 0.647 % (618) | 0.524 % (572) | 0.221 % (452) |
|  | 0.127 % (786) | 1.145 % (524) | 0.404 % (742) | 0.271 % (369) | 0.836 % (359) |
|  | 0.418 % (718) | 0.321 % (624) | 0.264 % (757) | 0.373 % (268) | 0.244 % (410) |
|  | 0.683 % (879) | 0.548 % (730) | 0.524 % (382) | 0.621 % (322) | 0.400 % (500) |
|  | 0.371 % (539) | 0.181 % (553) | 0.297 % (337) | 0.226 % (443) | 0.885 % (452) |
|  | 0.382 % (523) | 1.132 % (265) | 0.211 % (475) |  |  |
| Mean | 0.396 % | 0.579 % | 0.391 % | 0.403 % | 0.517 % |
| SD | ± 0.176 % | ± 0.455 % | ± 0.168 % | ± 0.167 % | ± 0.321 % |
| Significance | | T p = 0.379 | T p = 0.963 | T p = 0.946 | T p = 0.466 |
| Experiment 2  (24 h exposure + 0 h recovery) | 0.545 % (918) | 0.473 % (634) | 0.322 % (621) | 0.342 % (584) | 0 % (272) |
|  | 0.545 % (918) | 0.225 % (889) | 0.355 % (845) | 0.413 % (727) | 0.920 % (435) |
|  | 0.245 % (817) | 0.268 % (745) | 0.517 % (773) | 0.717 % (558) | 0.333 % (300 |
|  | 0.262 % (762) | 0.169 % (591) | 0.756 % (397) | 0.971 % (412) | 1.325 % (151) |
|  | 0.182 % (548) | 0.551 % (726) | 0.152 % (660) | 0 % (455) | 0 % (538) |
|  | 0.574 % (523) | 0 % (360) | 0.153 % (653) | 0.605 % (496) | 0.476 % (841) |
|  | 0.376 % (797) | 0.181 % (553) | 0.237 % (422) | 0.497 % (805) | 0.519 % (578) |
|  | 0 % (350) | 0.608 % (823) | 0.694 % (432) | 0.585 % (684) | 0.684 % (585) |
|  | 0.214 % (468) | 0.591 % (677) | 1.015 % (394) | 0.668 % (599) | 0.204 % (490) |
|  | 0 % (594) | 0.412 % (486) | 0.860 % (465) | 0.305 % (328) | 0.203 % (493) |
|  | 0.207 % (483) | 0.279 % (358) | 0.643 % (622) | 0.542 % (369) | 0.588 (340) |
|  | 0.272 % (368) | 0 % (536) |  |  |  |
| Mean | 0.285 % | 0.313 % | 0.518 % | 0.513 % | 0.477 % |
| SD | ± 0.194 % | ± 0.213 % | ± 0.296 % | ± 0.252 % | ± 0.400 % |
| Significance | | T p = 0.740 | R p = 0.090 | T p = 0.023 | R p = 0.339 |
| Experiment 2  (24 h exposure + 24 h recovery) | 0.406 % (1233) | 0.283 % (1061) | 0.485% (1030) | 0.651% (922) | 0.239% (419) |
|  | 0.569 % (1055) | 0.156% (647) | 0.459% (653) | 0.950% (421) | 0.920% (435) |
|  | 0.110 % (908) | 0.909% (770) | 0.738% (1219) | 0.519% (964) | 0.700% (714) |
|  | 0.679 % (589) | 0.489% (1023) | 0.536% (746) | 0.420% (715) | 1.471% (408) |
|  | 0.578 % (1038) | 0.571% (701) | 0.467% (642) | 0.385% (519) | 0% (272) |
|  | 0.112 % (891) | 0.435% (689) | 0.405% (741) | 0.489% (818) | 1.173% (341) |
|  | 0.283 % (1414) | 0.213% (939) | 0.694% (720) | 0.522% (575) | 0.653% (1226) |
|  | 0.636 % (629) | 0.145% (690) | 0.725% (1379) | 0.870% (460) | 0.699% (1001) |
|  | 0.604 % (828) | 0.452% (663) | 0.402% (747) | 0.829% (724) | 0.515% (582) |
|  | 0.182 % (1101) | 0.556% (902) | 0.575% (695) | 0.423% (710) | 1.141% (263) |
|  | 0.507 % (789) | 0.472% (636) | 0.629% (795) | 0.778% (643) | 0.906% (331) |
|  | 0.293 % (1025) | 0.292% (685) | 0.390% (1026) | 0.647% (309) | 0.935% (535) |
| Mean | 0.413 % | 0.414 % | 0.542 % | 0.623 % | 0.779 % |
| SD | ± 0.210 % | ± 0.216 % | ± 0.128 % | ± 0.210 % | ± 0.407 % |
| Significance | | T p = 0.991 | R p = 0.214 | T p = 0.018 | T p = 0.011 |

Table S13: Micronucleus frequencies assessed after cannabidiol treatment for 24 h or 24 h with additional 24 h of recovery (number of examined cells in parentheses)

| Cannabidiol | control | 0.159 µM | 0.318 µM | 1.590 µM | 3.180 µM |
| --- | --- | --- | --- | --- | --- |
| Experiment 1  (24 h exposure + 0 h recovery) | 0.691 % (579) | 0.262 % (764) | 0.522 % (575) | 0.347 % (577) | 1.394 % (574) |
|  | 0.622 % (643) | 0.356 % (843) | 0.667 % (750) | 0.416 % (481) | 0.347 % (577) |
|  | 0 % (553) | 0.254 % (787) | 0.401 % (748) | 0.341 % (586) | 0.315 % (635) |
|  | 0 % (699) | 0.620 % (645) | 0.565 % (708) | 0.371 % (809) | 0.423 % (473) |
|  | 0.266 % (752) | 0.256 % (781) | 0.736 % (679) | 0.340 % (588) | 0.438 % (457) |
|  | 0.563 % (533) | 0.372 % (807) | 0.450 % (667) | 0 % (487) | 1.329 % (602) |
|  | 0.175 % (572) | 0.245 % (817) | 0.307 % (652) | 0.635 % (630) | 0.509 % (589) |
|  | 0.549 % (728) | 0.906 % (773) | 0.638 % (627) | 0.139 % (718) | 0.729 % (686) |
|  | 0.267 % (750) | 0.274 % (730) | 0.429 % (700) | 0.860 % (465) | 0.323 % (619) |
|  | 0.153 % (654) | 0.707 % (707) | 0.181 % (552) | 0.323 % (619) | 0.384 % (521) |
|  | 0.295 % (678) | 0.612 % (654) | 0.300 % (666) | 0.345 % (579) | 0.365 % (548) |
|  | 0.347 % (577) | 0.381 % (525) | 0.431 % (696) | 0.571 % (525) | 0.600 % (500) |
| Mean | 0.324 % | 0.430 % | 0.474 % | 0.382 % | 0.605 % |
| SD | ± 0.234 % | ± 0.220 % | ± 0.164 % | ± 0.222 % | ± 0.378 % |
| Significance | | T p = 0.249 | T p = 0.1 | T p = 0.502 | R p = 0.043 |
| Experiment 1  (24 h exposure + 24 h recovery) | 0.432 % (694) | 0.510 % (981) | 0 % (731) | 0.830 % (723) | 0.772 % (777) |
|  | 0.577 % (866) | 0.257 % (1169) | 0.204 % (981) | 0.737 % (678) | 0.140 % (716) |
|  | 0.137 % (732) | 0.293 % (1023) | 0.304 % (987) | 0.653 % (766) | 0 % (775) |
|  | 0.219 % (915) | 0.365 % (821) | 0.650 % (923) | 0.292 % (1028) | 0.468 % (641) |
|  | 0.404 % (991) | 0.196 % (1020) | 0.338 % (888) | 0.362 % (829) | 0.334 % (599) |
|  | 0.277 % (722) | 0.581 % (1032) | 0.566 % (884) | 0.311 % (644) | 1.432 % (768) |
|  | 0.419 % (716) | 0.853 % (1055) | 0.238 % (842) | 0.614 % (814) | 1.170 % (769) |
|  | 0.529 % (945) | 0.303 % (989) | 0.123 % (810) | 0.411 % (974) | 1.018 % (884) |
|  | 0.628 % (955) | 0.835 % (958) | 0.442 % (904) | 1.133 % (618) | 1.264 % (870) |
|  | 0.424 % (943) | 0.438 % (914) | 0.873 % (687) | 0.369 % (813) | 0.303 % (661) |
|  | 0.332 % (903) | 0.459 % (872) | 0.796 % (879) | 0.244 % (821) | 0.528 % (757) |
|  | 0.143 % (698) | 0.737 % (678) | 0.772 % (907) | 0.731 % (684) | 0.625 % (640) |
| Mean | 0.387 % | 0.478 % | 0.441 % | 0.532 % | 0.700 % |
| SD | ± 0.161 % | ± 0.224 % | ± 0.287 % | ± 0.271 % | ± 0.463 % |
| Significance | | T p = 0.186 | R p = 0.624 | T p = 0.06 | R p = 0.126 |

Intralabratory micronucleus test

Table S14: Micronucleus frequencies after colchicine treatment assessed by two independent experimentators (number of examined cells in parentheses)

| Experiment 4 (24 h + 0 h) Experimentator 1 | control | 0.031 µM | 0.062 µM | 0.125 µM | 0.188 µM |
| --- | --- | --- | --- | --- | --- |
|  | 0.230 % (868) | 0.664 % (602) | 0.852 % (352) | 1.761 % (284) | 0.990 % (101) |
|  | 0.409 % (733) | 1.370 % (146) | 0.658 % (456) | 0.962 % (312) | 1.156 % (173) |
|  | 0.179 % (560) | 0.388 % (258) | 0.380 % (263) | 2.069 % (145) | 2.604 % (192) |
|  | 0.414 % (724) | 0.426 % (235) | 0 % (259) | 1.439 % (139) | 1.887 % (212) |
|  | 0.190 % (526) | 1.186 % (506) | 0.637 % (157) | 1.250 % (400) | 1.220 % (82) |
|  | 0.503 % (596) | 0.561 % (535) | 0.402 % (498) | 1.190 % (336) | 0.901 % (222) |
|  | 0.540 % (556) | 0.395 % (253) | 0.765 % (392) | 1.852 % (216) | 2.674 % (187) |
|  | 0.233 % (429) | 0.472 % (424) | 0.725 % (414) | 1.005 % (199) | 1.079 % (278) |
|  | 0.563 % (355) | 0.452 % (442) | 0.408 % (245) | 1.124 % (89) | 3.315 % (181) |
|  | 0 % (248) | 0.906 % (331) | 2.367 % (169) | 0.521 % (192) | 1.603 % (312) |
|  | 0.923 % (325) | 0.348 % (287) |  |  | 0.347 % (288) |
| Mean | 0.380 % | 0.652 % | 0.719 % | 1.317 % | 1.616 % |
| SD | ± 0.252 % | ± 0.350 % | ± 0.630 % | ± 0.469 % | ± 0.907 % |
| Significance | | R p = 0.088 | R p = 0.139 | T p < 0.001 | R p < 0.001 |
| Experiment 4 (24 h + 0 h) Experimentator 2 | 0.328 % (1831) | 0.272 % (736) | 0.164 % (611) | 0.458 % (655) | 2.609 % (230) |
|  | 0.285 % (1754) | 0.361 % (554) | 0.635 % (787) | 1.887 % (530) | 1.948 % (308) |
|  | 0.183 % (1637) | 0.208 % (480) | 0.255 % (783) | 2.079 % (529) | 1.775 % (338) |
|  | 0.376 % (1330) | 0.173 % (579) | 0.140 % (712) | 1.606 % (436) | 1.881 % (319) |
|  | 0.082 % (1226) | 0.291 % (688) | 0.525 % (381) | 1.700 % (353) | 2.069 % (290) |
|  | 0.193 % (1037) | 0.129 % (778) | 1.015 % (394) | 1.731 % (520) | 2.570 % (467) |
|  | 0.434 % (1153) | 0.313 % (639) | 0.266 % (751) | 0.438 % (457) | 1.578 % (507) |
|  | 0.295 % (1017) | 0.231 % (433) | 0.851 % (940) | 0.348 % (574) | 0.260 % (385) |
|  | 0.179 % (558) | 0.131 % (764) | 0.262 % (763) | 1.199 % (417) | 2.376 % (505) |
|  | 0.353 % (566) | 0.434 % (692) | 0.760 % (658) | 4.070 % (344) | 1.695 % (472) |
|  | 0.644 % (466) | 0.412 % (485) | 1.676 % (358) | 1.736 % (288) | 2.187 % (503) |
|  | 0.992 % (504) | 0.512 % (384) | 0.833 % (480) | 0.400 % (250) | 3.265 % (490) |
| Mean | 0.362 % | 0.290 % | 0.615 % | 1.471 % | 2.018 % |
| SD | ± 0.246 % | ± 0.124 % | ± 0.450 % | ± 1.048 % | ± 0.729 % |
| Significance | | R p = 0.583 | R p = 0.285 | R p < 0.001 | R p < 0.001 |

Table S15: Interlaboratory experiments from RWTH Aachen with cyclophosphamide and aflatoxin B1. The mean micronucleus frequencies were assessed for both compounds with either rat-derived S9 or ewoS9R for 3 h exposure and 45 h of recovery

| Experiment  RWTH Aachen with Aflatoxine B1 | control | 0.00625 mM | 0.0125 mM | 0.025 mM | 0.05 mM | 0.1 mM | 0.2 mM | 0.4 mM | 0.8 mM | 1.6 mM |
| --- | --- | --- | --- | --- | --- | --- | --- | --- | --- | --- |
| AFB1 + ewoS9R (3 h exposure + 45 h recovery)  Approach 1 | 0.4 % | 0.7 % | 0.5 % | 0.9 % | 1.0 % | 1.4 % | 1.4 % | 2.3 % | 5.2 % | 5.0 % |
| Approach 2 | 0.2 % | 0.6 % | 0.4 % | 1.0 % | 1.1 % | 1.2 % | 1.5 % | 2.1 % | 5.3 % | 4.5 % |
| AFB1 + S9 (3 h exposure + 45 h recovery)  Approach 1 | 0.8 % | 1.2 % | 1.0 % | 1.6 % | 1.5 % | 1.6 % | 1.9 % | 2.5 % | 3.1 % | 2.6 % |
| Approach 2 | 0.6 % | 1.4 % | 1.2 % | 1.4 % | 1.5 % | 1.7 % | 2.0 % | 2.2 % | 3.0 % | 2.4 % |
|  | control | 1.13 µM | 2.26 µM | 4.53 µM | 9.06 µM | 18.13 µM | 36.25 µM | 72.5 µM | 145 µM | 290µM |
| CP + ewoS9R (3 h exposure + 45 h recovery)  Approach 1 | 0.5 % | 0.5 % | 0.7 % | 0.7 % | 0.8 % | 0.9 % | 0.8 % | 0.8 % | 1.0 % | 1.2 % |
| Approach 2 | 0.6 % | 0.4 % | 0.5 % | 0.5 % | 0.8 % | 0.8 % | 0.7 % | 0.9 % | 0.9 % | 0.8 % |
| CP + S9 (3 h exposure + 45 h recovery)  Approach 1 | 0.8 % | 0.9 % | 0.6 % | 0.9 % | 0.4 % | 1.0 % | 1.5 % | 1.0 % | 1.2 % | 1.9 % |
| Approach 2 | 0.7 % | 0.6 % | 0.7 % | 0.7 % | 0.6 % | 0.9 % | 1.4 % | 1.1 % | 1.3 % | 1.8 % |

Table S16: Literature data of MN frequencies for different cell lines treated with 4-nitroquinoline and colchicine. The cells were investigated by the following authors HepG2 (Valentin-Severin 2003), VH-16 (Antoccia 1993), CHL (Schmuck 1988).

| **Chemical agent** | **CHL/IU** | **HepG2** | **VH-16** | **KCB** |
| --- | --- | --- | --- | --- |
| **4-NQO 0.05 µM** |  |  |  | 1.7 % |
| **4-NQO 0.1 µM** |  | 2 % |  | 2.3 % |
| **4-NQO 0.25 µM** |  | 2.2 % |  |  |
| **4-NQO 1 µM** |  | 3 % |  |  |
| **4-NQO 2.26 µM** | 5.90 % |  |  |  |
| **Colchicine 0.06 µM** |  |  |  | 1.5 % |
| **Colchicine 0.19 µM** |  |  |  | 2.0 % |
| **Colchicine 1.5 µM** |  |  | 2 % |  |
| **Colchicine 1.75 µM** |  |  | 2.7 % |  |
| **Colchicine 2 µM** |  |  | 3 % |  |

Nucleoplasmic bridges

Table S17: Frequencies of nucleoplasmic bridges assessed after cannabidiol treatment for 24 h or 24 h with additional 24 h of recovery (number of examined cells in parentheses)

| Cannabidiol | control | 0.159 µM | 0.318 µM | 1.590 µM | 3.180 µM |
| --- | --- | --- | --- | --- | --- |
| Experiment 1  (1 h) | 0 % (579) | 0.393 % (764) | 0.696 % (575) | 1.213 % (577) | 1.220 % (574) |
|  | 0.311 % (643) | 0.356 % (843) | 1.067 % (750) | 1.247 % (481) | 0.520 % (577) |
|  | 0.181 % (553) | 0.254 % (787) | 1.203 % (748) | 0.683 % (586) | 0.787 % (635) |
|  | 0.143 % (699) | 0.465 % (645) | 1.130 % (708) | 0.618 % (809) | 1.268 % (473) |
|  | 0.266 % (752) | 0.128 % (781) | 0.884 % (679) | 0.170 % (588) | 0.875 % (457) |
|  | 0.188 % (533) | 0.743 % (807) | 1.049 % (667) | 0.821 % (487) | 1.163 % (602) |
|  | 0.175 % (572) | 0.612 % (817) | 0.613 % (652) | 1.429 % (630) | 1.698 % (589) |
|  | 0.137 % (728) | 1.035 % (773) | 0.957 % (627) | 0.279 % (718) | 1.458 % (686) |
|  | 0 % (750) | 0.959 % (730) | 1.143 % (700) | 1.075 % (465) | 1.131 % (619) |
|  | 0.153 % (654) | 1.132 % (707) | 0.725 % (552) | 0.969 % (619) | 0.960 % (521) |
|  | 0.147 % (781) | 1.376 % (654) | 1.201 % (666) | 1.382 % (579) | 2.007 % (548) |
|  | 0.520 % (807) | 0.762 % (525) | 0.575 % (696) | 0.952 % (525) | 1.200 % (500) |
| Mean | 0.181 % | 0.668 % | 0.948 % | 0.878 % | 1.195 % |
| SD | ± 0.138 % | ± 0.385 % | ± 0.232 % | ± 0.407 % | ± 0.401 % |
| Significance | | R p = 0.001 | R p < 0.001 | R p < 0.001 | R p < 0.001 |
| Experiment 1  (24 h) | 0 % (694) | 0.306 % (981) | 0.821 % (731) | 0.415 % (723) | 0.772 % (777) |
|  | 0.231 % (866) | 0.513 % (1169) | 0.408 % (981) | 0.295 % (678) | 1.117 % (716) |
|  | 0.273 % (732) | 0.880 % (1023) | 0.203 % (987) | 0.522 % (766) | 0.774 % (775) |
|  | 0.109 % (915) | 0.244 % (821) | 0.108 % (923) | 0.584 % (1028) | 0.780 % (641) |
|  | 0 % (991) | 0.392 % (1020) | 0.788 % (888) | 0.724 % (829) | 0.835 % (599) |
|  | 0.139 % (722) | 0.388 % (1032) | 0.566 % (884) | 1.087 % (644) | 0.651 % (768) |
|  | 0.279 % (716) | 0.379 % (1055) | 0.475 % (842) | 1.106 % (814) | 1.430 % (769) |
|  | 0.423 % (945) | 0.303 % (989) | 0.864 % (810) | 0.411 % (974) | 0.905 % (884) |
|  | 0.628 % (955) | 0.418 % (958) | 0.332 % (904) | 0.485 % (618) | 0.460 % (870) |
|  | 0.212 % (943) | 0.656 % (914) | 0.437 % (687) | 0.738 % (813) | 0.605 % (661) |
|  | 0.775 % (903) | 0.344 % (872) | 0.455 % (879) | 0.731 % (821) | 1.057 % (757) |
|  | 0.430 % (698) | 0.737 % (678) | 0.221 % (907) | 0.585 % (684) | 0.938 % (640) |
| Mean | 0.298 % | 0.460 % | 0.461 % | 0.639 % | 0.858 % |
| SD | ± 0.238 % | ± 0.196 % | ± 0.248 % | ± 0.254 % | ± 0.257 % |
| Significance | | T p = 0.663 | T p = 0.081 | T p = 0.002 | T p < 0.001 |

Table S18: Comparison of the workload of the *in vitro* micronucleus test with the KCB H2B-eGFP cell line and standardised protocols

|  | DIN with V79 cells | KCB H2B-eGFP | OECD 487 |
| --- | --- | --- | --- |
| Cultivation temp | 37°C | 26°C | variable |
| Preparation of cell culture media | 10 min | 10 min | 10 min |
| Preparation of test compounds without S9 fraction | 30 min | 30 min | 30 min |
|  |  |  |  |
| Metabolic activation | 30 min | 60 min (with pre-incubation due to lower incubation temp. 26°C vs 37°C, required for S9 enzymes) | 30 min |
| Thawing of the cells and pre-culture | 60 min | Thawing of cells not necessary due to suitability of multi-passages culture (also not needed if cell coated plates are used) | 60 min |
| Cell transfer to vessels used in the test | 10 min | 10 min (Not needed if cell coated plates are used) | 10 min |
| Cell-coated plates  Removal of sealing mat and replacement of transport medium | Not applicable | 5 min | Not applicable |
| Exposure of the cells to the test compound | 10 min | 10 min | 10 min |
| Preparation of the cells for imaging | 60 min | 5 min (test medium replacement) | 60 min |
| Dye | 40 min | Not needed self-fluorescent cells | Variable depending on the dye used |
| Analysis and statistical evaluation | 3 h | 2 h with automatised live-imaging | 3 h |
| Total time | 6 h 40 min (with metabolic activation7 h 10 min) | 3 h 10 min (with metabolic activation 4 h 10 min) | 6 h 40 min (with metabolic activation 7 h 10 min) |
| Total time with cell coated plates | Not available | 3 h (with metabolic activation 4 h) | Not available |
